# The castration-resistant prostate cancer-associated SNP rs11067228 facilitates neuroendocrine differentiation through an enhancer-mediated chromatin interaction with SRRM4

**DOI:** 10.7150/ijbs.124731

**Published:** 2026-01-08

**Authors:** Yuan Jiang, Zhenhao Zhao, Peng Li, Guangsong Su, Yuyang Qian, Yuting Zhao, Bo Wang, Yunlong Bai, Lei Zhang, Zhongfang Zhao, Jiandang Shi, Wange Lu

**Affiliations:** 1 State Key Laboratory of Medicinal Chemical Biology, College of Life Sciences, Nankai University, 300071 Tianjin, People's Republic of China; 2 Department of Laboratory Medicine and Institute of Precise Medicine, The First Affiliated Hospital, Sun Yat-sen University, 510080 Guangzhou, Guangdong, People's Republic of China; 3 Weifang People's Hospital, Shandong Second Medical University, 261000 Weifang, Shandong, People's Republic of China

**Keywords:** SNP rs11067228, enhancer, long-range chromatin interaction, neuroendocrine differentiation, prostate cancer

## Abstract

Neuroendocrine prostate cancer is an aggressive disease characterized by early metastasis, drug resistance and poor prognosis. Genome-wide association studies (GWAS) previously identified numerous single nucleotide polymorphisms (SNPs) associated with prostate cancer. SNP rs11067228 as a significant variant associated with castration-resistant metastasis (CM) in prostate cancer (PCa). However, mechanisms underlying activity of the rs11067228 risk variant remain unclear. Here, we demonstrated that risk SNP rs11067228 is located in an H3K27ac-enriched active enhancer, and that activity of that region affects castration-resistance and neuroendocrine differentiation in PCa cells. We identified the RNA-splicing factor *SRRM4* as a functional target gene as shown in both cell line and xenograft model. In addition, overexpression of *SRRM4* is sufficient to induce PCa cell drug resistance and neuroendocrine differentiation. Moreover, site-directed mutation of the rs11067228 non-risk G to the risk A allele enabled binding of the transcription factor SOX4, activating candidate target gene expression. Taken together, our findings indicated that the rs11067228-associated enhancer modulates expression of *SRRM4* via allele-specific long-range chromatin interactions, thereby governing PCa drug resistance and neuroendocrine differentiation.

## Introduction

Prostate cancer (PCa) is a leading cause of cancer-related mortality in men worldwide. At present, androgen deprivation therapy (ADT) is the first-line treatment for advanced or metastatic PCa. Next-generation inhibitors of androgen receptor (AR) signaling such as enzalutamide has successfully improved patient survival. However, most patients eventually develop resistance to ADT, and their tumors will progress to castration-resistant prostate cancer (CRPC) within 2-3 years 1, 2. A subset of CRPC, known as neuroendocrine prostate cancer (NEPC) emerges at the lineage transition from adenocarcinoma to neuroendocrine cells and is induced by anti-androgen therapy. NEPC is characterized by the acquisition of neuroendocrine features (e.g., expression of *SYP (*synaptophysin),* CHGA (*chromogranin A) and *CHGB (*chromogranin B)), leading to resistance to conventional anti-androgen therapies and a median survival of less than 1 year 3-9. Moreover, PCa etiology depends on many factors, such as genetics, androgen and vitamin D, and others. Among these factors, genetics rank first and is a factor in ~42% of PCa cases^10^. Thus, understanding the genetic basis of PCa and its resistance to ADT is essential for the development of targeted therapies and personalized treatment strategies.

Analysis of genome-wide association studies (GWAS) has served as an effective strategy to investigate complex genetic diseases. At present, advances in GWAS analysis have identified >200 independent single-nucleotide polymorphisms (SNPs) associated with genetic risk of PCa 11-22. However, a significant number of these SNPs have an undetermined function. A major challenge in the post-GWAS era is to define intrinsic molecular mechanisms linking genetic risk factors with PCa. Interestingly, a few risk SNPs, such as rs11672691 and rs887391^23^, have been verified as associated with PCa progression. Moreover, PCa incidence is highest among men of African ancestry 24, and GWAS studies have shown that rs11067228 is associated with PCa risk in African-American men 25, 26. It is also reported that rs11067228 is significantly associated with castration-resistant metastasis (CM) based on multivariable analysis (P < 0.05) 27. However, mechanisms underlying how rs11067228 regulates PCa development and CM remain elusive.

Analyses of three-dimensional chromatin architecture have revealed that regulatory elements orchestrate long-range chromatin interactions to either activate or repress gene expression 28-32. While some SNPs are located in gene exons and alter function of proteins encoded by those alleles, others reside in non-coding regions and likely regulate transcription. Such variants may contribute to formation of specific transcription factor (TF) motifs or regulatory elements and to regulate gene expression via long-range chromatin interactions 28, 33, 34. For example, the risk SNP rs7463708, positioned on the distal enhancer of the *PCAT1* gene, specifically recruits the TF ONECUT*2* to regulate expression of the lncRNA* PCAT1* in the context of PCa 35. Moreover, we previously found that a risk SNP rs55958994-related enhancer located in a *KRT8* intron regulates expression of genes such as *CNTN1* via long-range chromatin interactions to promote PCa progression 36. Accordingly, we hypothesized that SNP rs11067228 may regulate genes crucial to PCa via long-range chromatin interactions and potentially impact PCa castration-resistance.

Here, we report that SNP rs11067228 is located in an active enhancer in PCa cells. Functional assays revealed a significantly decreased in cell migration and colony formation after deletion of the enhancer region. Strikingly, we found that this risk enhancer is associated PCa cell drug sensitivity and neuroendocrine differentiation, based on analysis of RNA sequencing (RNA-seq) data in the Kyoto Encyclopedia of Genes and Genomes (KEGG). Moreover, we conducted circular chromosome conformation capture (4C) to define the interactome of the risk enhancer. Interestingly, we identified *SRRM4*, which encodes an RNA-splicing factor, as a key regulator implicated in PCa castration-resistance and neuroendocrine differentiation. Also, conversion of rs11067228 from the non-risk G to the risk A allele in-situ increased tumor cell colony formation and invasive migration, and the risk A allele promoted drug resistance and PCa cell neuroendocrine differentiation. The risk A allele also bound the TF SOX4*,* activating expression of its target genes. Overall, these findings indicated that the PCa risk SNP rs11067228-related enhancer contributes to castration-resistance and neuroendocrine differentiation by regulating *SRRM4* through long-range chromatin interaction. This work reveals a potential target potentially useful both for more precise diagnosis and more effective treatment.

## Materials and Methods

### Study approval

All the experimental procedures involving mice in this study were approved by Nankai University Ethics Committee (approval no.2024-SYDWLL-000655). All animal housing and experiments were conducted in strict accordance with the institutional guidelines for Care and Use of Laboratory Animals and were approved by the Nankai University Laboratory Animal Use and Care Committee.

### Cell lines

The human PCa cell lines 22Rv1, LNCaP and C4-2B were obtained from the American Type Culture Collection (ATCC). Cells were incubated at 37°C with 5% CO2, and cultured in 10% (v/v) fetal bovine serum (FBS) and 90% RPMI- 1640 medium (Gibco) supplemented with 1% penicillin/streptomycin (Gibco). HEK-293T cells were obtained from the ATCC, incubated at 37°C with 5% CO2 and cultured with 10% (v/v) FBS and 90% Dulbecco's Modified Eagle Medium supplemented with 1% penicillin/streptomycin (Gibco). During this study all cell lines remained free of mycoplasma.

### Chromatin immunoprecipitation (ChIP)

1 × 10^7^ 22Rv1 cells were cross-linked with 1% formaldehyde and quenched in 0.125 M glycine. Cell pellets were harvested in Farnham lysis buffer [50 mM Tris-HCl (pH 7.5), 150 mM NaCl, 0.5% NP-40, 5 mM EDTA, 1% Triton X-100] supplemented with 1 mM phenylmethylsulfonyl fluoride (PMSF) and 1× protease inhibitor cocktail and incubated 30 min on ice to isolate nuclei. Nuclei were suspended in SDS lysis buffer [1% SDS, 50 mM Tris-HCl, 10 mM EDTA and 1× protease inhibitor cocktail] and sonicated to shear chromatin to DNA fragment sizes of 100-500 base pairs (bp). Supernatants were collected by centrifuging at 12,000 rcf for 10 mins at 4°C and precleared 1 h with 50 μl protein G agarose beads (Invitrogen). 2% of the precleared sample served as input, and the remainder was immunoprecipitated with 5 μg H3K27ac antibody (ab4729, Abcam) bound to 50 μl protein G agarose beads at 4°C overnight. Beads were washed 5 times with LiCl IP washing buffer [100 mM Tris-HCl (pH7.5), 500 mM LiCl, 1% NP-40 and 1% sodium deoxycholate] and cross-links were reversed in buffer containing Proteinase K overnight at 65°C. Finally, DNA was purified using a PCR Purification Kit (Qiagen) and eluted in 30 μl H_2_O. Precipitated and input DNA were sequenced using an Illumina HiSeq4000 sequencer platform. Reads were mapped to the GRCh37/hg19 reference genome using HISAT2 37 and further analyzed with the HOMER (Hypergeometric Optimization of Motif Enrichment) package 38.

### Soft agar assays

Culture medium (1.5 ml) containing 1.2% SeaPlaque^TM^ agarose (Lonza) was plated into 6-well plates and allowed to solidify at 4°C. Agarose at a final concentration of 0.7% and containing 5000 22Rv1 cells was carefully plated on top of the solidified GTG agarose. Plates were then incubated at 37°C with 5% CO_2_, and the medium was changed every 3 days. After 2-3 weeks, colonies were stained in 0.005% crystal violet in 4% paraformaldehyde solution and counted.

### Transwell assays

The upper chambers of Transwell plates (8 μm; Corning Costar) were precoated with diluted Matrigel (Corning). 22Rv1 cells were trypsin-digested and resuspended in serum-free medium. 200 μl of the cell suspension (2 × 104 cells per chamber) was seeded into Transwell plates, and 500 μl of complete RPMI 1640 Medium was added into the lower chambers. After 48 hours of incubation, invasive cells that had migrated to lower chambers were stained in 0.1% crystal violet and counted under a microscope.

### Lactate dehydrogenase assay

Lactate dehydrogenase in the culture medium was evaluated using the LDH cytotoxicity kit (Promega). In brief, cells were cultured in 96-well plates and then treated with enzalutamide (100 μM). The culture medium was collected at 24 and 48 h, and LDH activity was assayed in accordance with the manufacturer's instructions. The OD 490 nm was measured using a microplate reader. Levels of released LDH in each group were calculated as a percentage of the total amount (namely, the positive control as described in the kit protocol).

### MTS assay

2 × 103cells were cultured in 96-well plates and treated with enzalutamide (60 μM). 20 μl MTS solution was added to each well at times 6 h, 24 h, 48 h, 72 h or 96 h followed by 2 hours. The OD 490 nm was measured using a microplate reader.

### Luciferase reporter assays

The H3K27ac region surrounding rs11067228 (3787 bp, Chr12:115092472-115096258, hg19) was amplified from 22Rv1 cell genomic DNA and cloned into the pGL3 promoter reporter vector (E1761, Promega) upstream of the SV40 promoter. This region was chosen based on H3K27ac ChIP-seq data from 22Rv1 cell analysis. The SNP-mutated vector was established by site-directed mutagenesis. Enhancers with risk (A) or non-risk (G) alleles were individually cloned into the pGL3 vector. 22Rv1 cells were seeded into 24-well culture plates and cultured overnight. Reporter plasmids and the pRL-TK *Renilla* luciferase control vector (E2241, Promega) were then co-transfected into 22Rv1 cells using Lipofectamine 3000 reagent (Invitrogen). Cells were collected 36 h later, and luciferase activity determined using the Dual-Luciferase Reporter Assay System (E1910, Promega). Relative luminescent signals from experimental samples were normalized to *Renilla* signals.

### CRISPR/Cas9-mediated deletions and mutations

To delete the entire SNP rs11067228-associated enhancer-like region, 22Rv1 cells were transfected with plasmids containing Cas9 and guide RNAs targeting that region. Colonies were derived from single cells and tested for target region deletion. Cell clones were genotyped, and three clones with homozygous deletion of the rs11067228-containing enhancer region plus three control clones were used for RNA-seq analysis. Sequences of single guide RNAs and enhancer KO screening primers are listed in Table S1. For site-directed mutation of SNP rs11067228, we co-transfected 22Rv1 cells with two Cas9 plasmids [pSpCas9n(sgRNAs)] containing guide RNAs targeting the enhancer-like region, and DNA fragments containing the (A) allele as repair templates. A single-nicking strategy was applied to reduce undesirable off-target mutagenesis. Sequences of single guide RNAs and SNP genotyping primers are shown in Table S4. SgRNAs were designed using a CRISPR design tool (https://zlab.bio/guide- design-resources). The region containing the mutation was amplified using specific primers, and SNP genotyping was performed by Sanger sequencing.

### Lentivirus production

HEK-293T cells were seeded into a 10 cm plate and incubated overnight at 37 °C and 5% CO_2_. Cells were then transfected with 8 μg of targeting plasmid and 2 μg of pMD2.G (Addgene, 12259), and 5 μg of psPAX2 (Addgene, 12260) packaging plasmids using 24 μl of LipoFilter^TM^ (HANBIO, HB-TRLF-1000). After transfection for 48 h, virus supernatants were collected and centrifuged at 3,000*rcf* for 10 mins at 4°C to remove the debris. Supernatants were aliquoted and stored at -80 °C.

### CRISPRi assay

A stable 22Rv1 CRISPRi cell line was generated using the Lenti-dCas9-KRAB-blast plasmid (89567, Addgene). In brief, lentiviral particles were generated in HEK-293T cells using the pMDG.2 and psPAX2 packaging plasmids and then used to infect 22Rv1 cells for 6-8 hr. Cells were then selected 5-7 days in 5 μg/ml blasticidin. CRISPRi sgRNAs sequences targeting genes regulated by the rs11067228-related enhancer were subsequently cloned into lentiGuide-Puro plasmid (52963, Addgene) using the restriction enzyme BsmBI. Lentiviral particles were generated, and stable CRISPRi cells were infected as described above and selected in 1 μg /ml puromycin for 2-3 days. RNA was then collected to assess gene expression levels. CRISPRi sgRNAs were designed using the GPP Web Portal (Broad institute; https://portals.broadinstitute.org/gpp/public/analysis-tools/sgrna-design). Sequences of single guide RNAs are shown in Table S7.

### CRISPRa assay

A stable CRISPRa cell line was generated using the lentiMPHv2 plasmid (89308, Addgene). Lentiviral particles were generated in HEK-293T cells using pMDG.2 and psPAX2 packaging plasmids. Enhancer-KO cells were infected for 6-8 hr and selected in 500 μg/ml hygromycin for 5-7 days. CRISPRa sgRNAs targeting genes regulated by the rs11067228-related enhancer were designed and cloned into lentiSAMv2 plasmid (75112, Addgene) using the restriction enzyme BsmBI, and packaged and infected as described above. Infected enhancer-KO CRISPRa cells were selected in 5 μg/ml blasticidin for 5-7 days and RNA was collected to assess gene expression levels. CRISPRa sgRNAs were designed using the GPP Web Portal (Broad institute; https://portals.broadinstitute.org/gpp/public/analysis-tools/sgrna-design). Sequences of single guide RNAs are provided in Table S7.

### Tumor xenograft model

1 × 107cells were suspended in PBS/Matrigel (1:1; Corning) and implanted into the right armpit of 4 weeks old female BALB/c nude mice (100 μL per mice). The volume of the tumor xenografts was measured using an electronic vernier caliper every 3 days after three weeks (calculated as Volume = 0.5 × Length × Width^2^). The mice were sacrificed at week 8 and tumors were harvested carefully and photographed.

### Circular chromatin conformation capture assay (4C) sequencing

The 4C assay was carried out using a published protocol 39 with some modifications. Briefly, 1 × 10^7^ cells were cross-linked in 1% formaldehyde at room temperature for 10 min, and then quenched by 0.125 M glycine. Nuclei were resuspended in 5 ml cold lysis buffer (10 mM Tris- HCl pH 7.5, 10 mM NaCl, 5 mM MgCl_2_, 10 mM EGTA, 0.2% NP-40 and 1 mM PMSF), incubated on ice for 10 min, and then digested with 400 U DpnII enzyme at 37°C with rotation, followed by addition of 4000 U T4 DNA ligase for in-situ ligation overnight at 16°C. Ligated DNA was de-crosslinked overnight using 30 μl of buffer containing Proteinase K (10 mg/ml) at 65°C overnight. Remaining RNA was removed by treatment with 30 μl RNase A (10 mg/ml) and incubation at 37 °C for 45 min. DNA was extracted with an equivalent volume of phenol/chloroform/isoamyl alcohol, and pellets were dissolved in 150 μl 10 mM Tris-HCl (pH 7.5). Purified DNA was digested with 100 U CviQI enzyme, which was then inactivated at 65 °C for 20 min and DNA was circularized by incubation 4 h with 4000 U T4 DNA ligase at room temperature. DNA was purified using phenol/chloroform and further purified using a QIAquick PCR purification kit. DNA concentration was determined using Qubit (Thermo Fisher). Circularized DNA was amplified with SNP-specific inverse primers (Table S6). Each 4C library was sequenced as 150 bp paired-end reads using the Illumina Hiseq 4000 system. Pair-end reads were split into two parts based on the SNP-specific primer. For each read-pair, the read with the reading primer was trimmed and digested in silico. Digested fragments were aligned to the reference genome using the BWA tool^40^. We then calculated coverage of every restriction fragment and converted values to reads per million (RPM).

### Chromosome conformation capture (3C) Assay

The 3C assay was performed in 22Rv1 cells as described 41. Briefly, 1 × 107cells were fixed in 1% formaldehyde and fractionated to obtain the nuclear fraction. Nuclear lysates were digested with 400 U HindIII overnight at 37°C followed by addition of 4000 U T4 DNA ligase for in-situ ligation at 16°C overnight. DNA was extracted with phenol/chloroform and interactions between the SNP rs11067228 locus and the candidate partners were detected by PCR. Sequences of primers used for the analysis are provided in Table S3.

### 3C-qPCR with genomic DNA-based PCR efficiency correction

To account for primer-specific amplification efficiencies, a control library was generated from genomic DNA. High molecular weight genomic DNA from parental 22Rv1 cells was digested to completion with the same restriction enzyme used for 3C. The digested DNA was ligated under highly dilute conditions (~ 2.5 ng/μL) to generate a library of random ligation products. This genomic DNA control library was serially diluted and used as a template to generate standard curves for each 3C-qPCR primer pair. Amplification efficiency (E) for each primer pair was calculated from the slope of the standard curve using the formula E = [10^(-1/slope)] - 1. Only primer pairs with efficiencies between 90% and 110% were used for analysis. Interaction frequencies were calculated from efficiency-corrected Ct values. The relative quantity (RQ) for each interaction was determined as RQ = (1+E)^(-Ct). RQ values were normalized to the RQ of the anchor self-ligation product to obtain the final interaction frequencies. Data are presented as mean ± SEM of three biological replicates.

### RNA isolation and RT-qPCR

Total RNA was isolated from cultured cells using TRIzol reagent. 1 μg RNA was reverse- transcribed to cDNA using the PrimeScript RT Reagent Kit with a gDNA eraser Kit (Takara, no. RRO47B). RT-qPCR was performed with qPCR SYBR Green Master mix (YEASEN) using the CFX96 (Bio-rad). Relative target gene expression was analyzed using the comparative Ct method and normalized to expression levels of the housekeeping gene HPRT1. Sequences of RT-qPCR primers used for analysis are shown in Table S2.

### RNA sequencing

Barcoded RNA-seq libraries were sequenced as 150-bp paired-end reads using the Illumina HiSeq 4000 platform. Reads were mapped to the reference genome (GRCh37/hg19) using HISAT2 37, 42 with a GENCODE GTF file supplied as gene model annotations. HTSeq 43 was used to quantitate transcript abundance for each gene. DESeq2 44 was used to perform normalization and regularized log transformations of read counts. PCA was conducted with regularized log-transformed data. Hierarchical clustering was performed over samples with replicates. Euclidean distance and complete linkage were used as a clustering metric and method, respectively. Lists of genes showing differential transcript abundance between enhancer KO and WT samples were compiled based (i) a Benjamini-Hochberg adjusted *P* < 0.05 and (ii) a log2fold-change in transcript abundance (enhancer KO versus WT) of >1.3 for up-regulated genes or <-1.3 for down-regulated genes. Differentially expressed genes were subjected to functional classification analysis using DAVID (Database for Annotation, Visualization and Integrated Discovery) version 6.8 45, 46. We used Gene Cluster software 3.0 (http://bonsai.hgc.jp/~mdehoon/software/cluster/software.htm) to perform clustering analysis of gene expression data. Genes were centered by means across samples, and hierarchical clustering was performed over genes and samples. The similarity metric of Euclidean distance and the clustering method of complete linkage were used. Clustering results were examined and visualized in Java TreeView (http://jtreeview.sourceforge.net/).

### Western blotting

Proteins were collected from cells using radioimmunoprecipitation assay buffer supplemented with a 1× protease inhibitor cocktail. Sample loading was based on results of a bicinchoninic acid assay. Protein lysates were separated by electrophoresis on 10% SDS/PAGE gels and transferred to PVDF membranes (0.45 μm; Millipore, Bedford, MA). Membranes were blocked and incubated with primary antibody overnight at 4°C, followed by incubation with secondary antibodies for 1 hour at room temperature. Bands on blots were detected by GelDoc XR+ (Bio-rad) using an enhanced chemiluminescence kit (Millipore). β-Tubulin served as a loading control. The same membrane was stripped and re-probed for the detection of different proteins. Stripping was performed using a mild stripping buffer (15 mM glycine, 0.1% SDS, 1% Tween 20, pH 2.2) for 15 minutes at room temperature with gentle agitation. Following stripping, the membrane was extensively washed in TBST before being re-blocked and incubated with the next primary antibody.

### DNA-protein pull-down assay

DNA-protein pull-down was performed as previously described 47. In brief, 22Rv1 SNP rs11067228-mutated cells were lysed by sonication in lysis buffer containing 50 mM Tris-HCl pH8.0, 100 mM KCl, 5 mM MgCl_2_, 1 mM DTT, 0.5% NP-40 and protease inhibitors to prepare nuclear extracts, which were then precleared 1 h with Streptavidin MagBeads (GenScript, L00424). Precleared supernatants were then incubated with 5′-biotinylated DNA probes and supplemented with 1 mM phenylmethylsulfonyl fluoride (PMSF) and a 1× protease inhibitor cocktail and incubated at 4 °C overnight. DNA-bound proteins were collected by incubating with Streptavidin MagBeads at 4 °C for 1 h. Beads were then washed five times with PBS, and bound proteins were released by boiling beads 5 min in 2 x protein loading buffer at 100 °C. Boiled protein samples were trypsin-digested for MS analysis. Sequences of biotinylated double-strand oligonucleotides used in the pull-down assay are shown in Table S8.

### Quantification and statistical analysis

All data was analyzed by t-test. Statistically significant p values are indicated in figures as follows: *** p<0.001, ** p<0.01, * p<0.05.

## Results

### The region containing SNP rs11067228 is a functional enhancer in PCa cells

**Figure 1:**
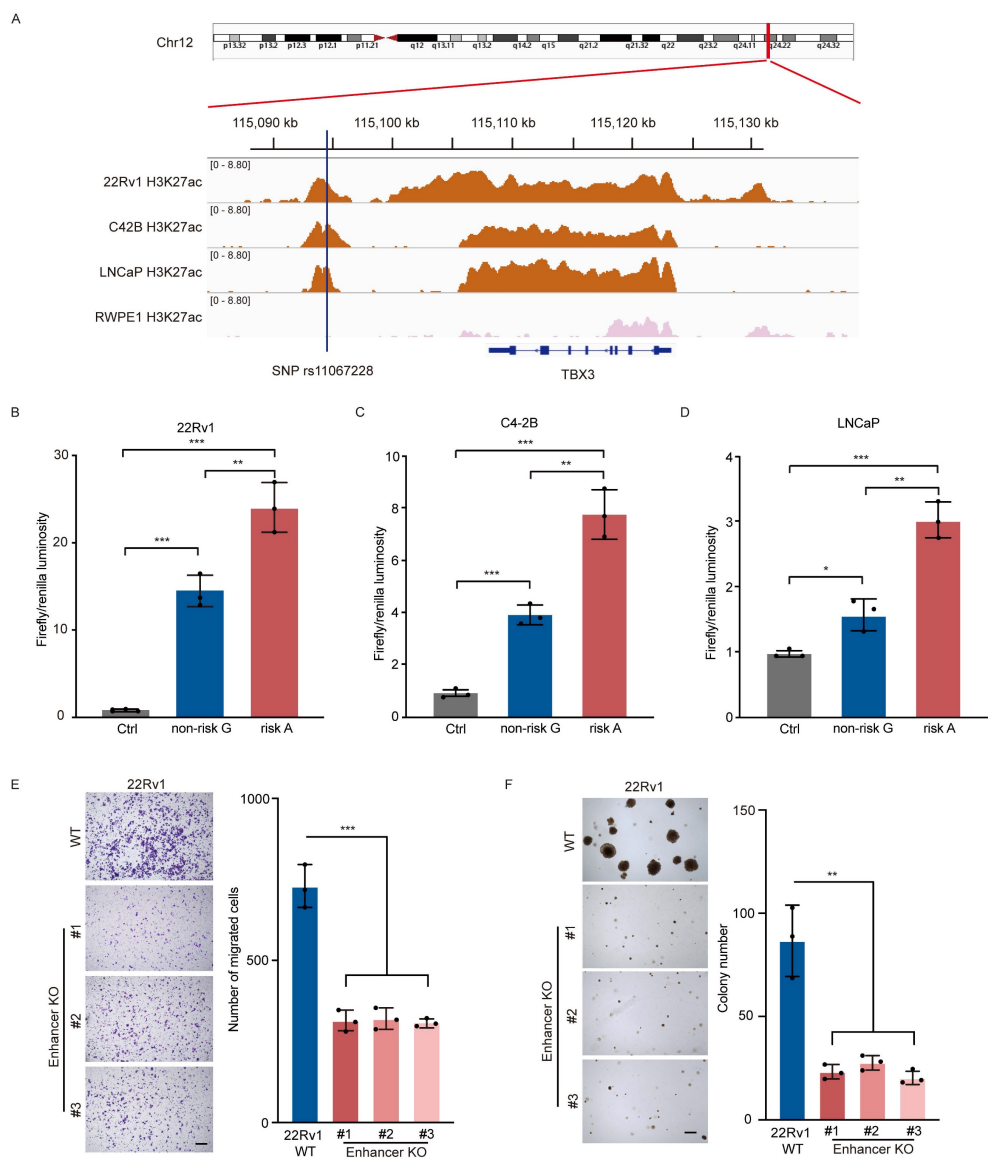
The rs11067228 locus is a functional enhancer in PCa cells. (A) H3K27ac ChIP-seq data of the rs11067228 locus in C4-2B, LNCaP and 22Rv1 PCa lines and in the normal prostate epithelial cell line RWPE1. (B, C, D) Assessment of luciferase activity in 22Rv1, C4-2B and LNCaP cells transduced with a reporter harboring either the non-risk (G) rs11067228-associated enhancer region or the risk (A) allele. The pGL3 promoter vector lacking the enhancer served as control. Firefly luciferase signals were normalized to Renilla signals. Data represent means ± S.E.M. of three independent experiments. ***P < 0.001, **P < 0.01, *P < 0.05. (E) Transwell assays of WT 22Rv1 cells and three enhancer KO lines. Cells migrating to the lower chambers were stained with 0.1% crystal violet (left). Scale bar =100 μm. Quantification of corresponding migrated cells is at right. Data represent means ± S.E.M. of three independent experiments. ***P < 0.001. (F) Analysis of colony formation in soft agar of WT 22Rv1 cells and three enhancer KO lines (left). Scale bar =100 μm. Quantification is at right. Data represent means ± S.E.M. of three independent experiments. **P < 0.01.

SNP rs11067228 is located at 12*q*24, a linkage disequilibrium block that contains the gene *TBX3* (T-Box Transcription Factor 3), and the corresponding risk allele (A) is associated with PCa risk in African American men 25, 26 and castration-resistant PCa metastasis (CM) 27. To investigate how the risk allele functions in PCa progression, we first performed chromatin immunoprecipitation followed by next-generation sequencing (ChIP-seq) in 22Rv1, C4-2B and LNCaP PCa lines for the active histone mark H3K27ac, as a means to identify enhancer-like elements. Significant H3K27ac peaks were notably enriched at the locus of the risk SNP, indicating that this non-coding region is an active enhancer in the three PCa lines investigated. Conversely, we observed no such signals when we performed comparable analysis of the normal prostate epithelial cell line RWPE1 (Fig. 1A). This finding indicates that the region containing SNP rs11067228 is a PCa-specific acquired active enhancer.

We then performed luciferase reporter assays to assess whether transcriptional activity is regulated differently by rs11067228 risk (A) and non-risk (G) alleles. To do so we cloned each allele-containing enhancer fragment separately into luciferase reporter plasmids and assessed luciferase activity in 22Rv1, C4-2B and LNCaP cells. Transduction of either the (A) or (G) allele enhanced luciferase activity relative to the control vector, which lacked the enhancer insertion, but the enhancer region harboring the risk (A) allele exhibited higher luciferase activity than did the (G) non-risk allele (Fig. 1B-D), suggesting that the presence of risk (A) allele of SNP rs11067228 increases enhancer activity in PCa cells.

Next, to assess the contribution of the rs11067228-associated enhancer to PCa progression, we narrowed our focus to 22Rv1 cells, which is a diploid cancer line and thus a good model for creation of homozygous deletions. Accordingly, we employed CRISPR-Cas9 technology to delete a ~4 kb enhancer fragment containing SNP rs11067228 in an intergenic region of chromosome 12 in 22Rv1 cells (Fig. S1). Then to determine how the risk SNP rs11067228-related enhancer functions in PCa progression, we characterized 3 homozygous deletion clones for invasive migration and colony formation (Fig. 1E, F). Relevant to migration, transwell assays showed that enhancer knockout (KO) cells exhibited significantly reduced invasive migration activity compared to 22Rv1 wild-type (WT) PCa cells (Fig. 1E). Moreover, soft agar assays demonstrated substantially decreased tumor cell colony formation in enhancer KO relative to WT cells (Fig. 1F).

### The risk SNP rs11067228-related enhancer is essential for the development and maintenance of PCa drug resistance and neuroendocrine differentiation

**Figure 2:**
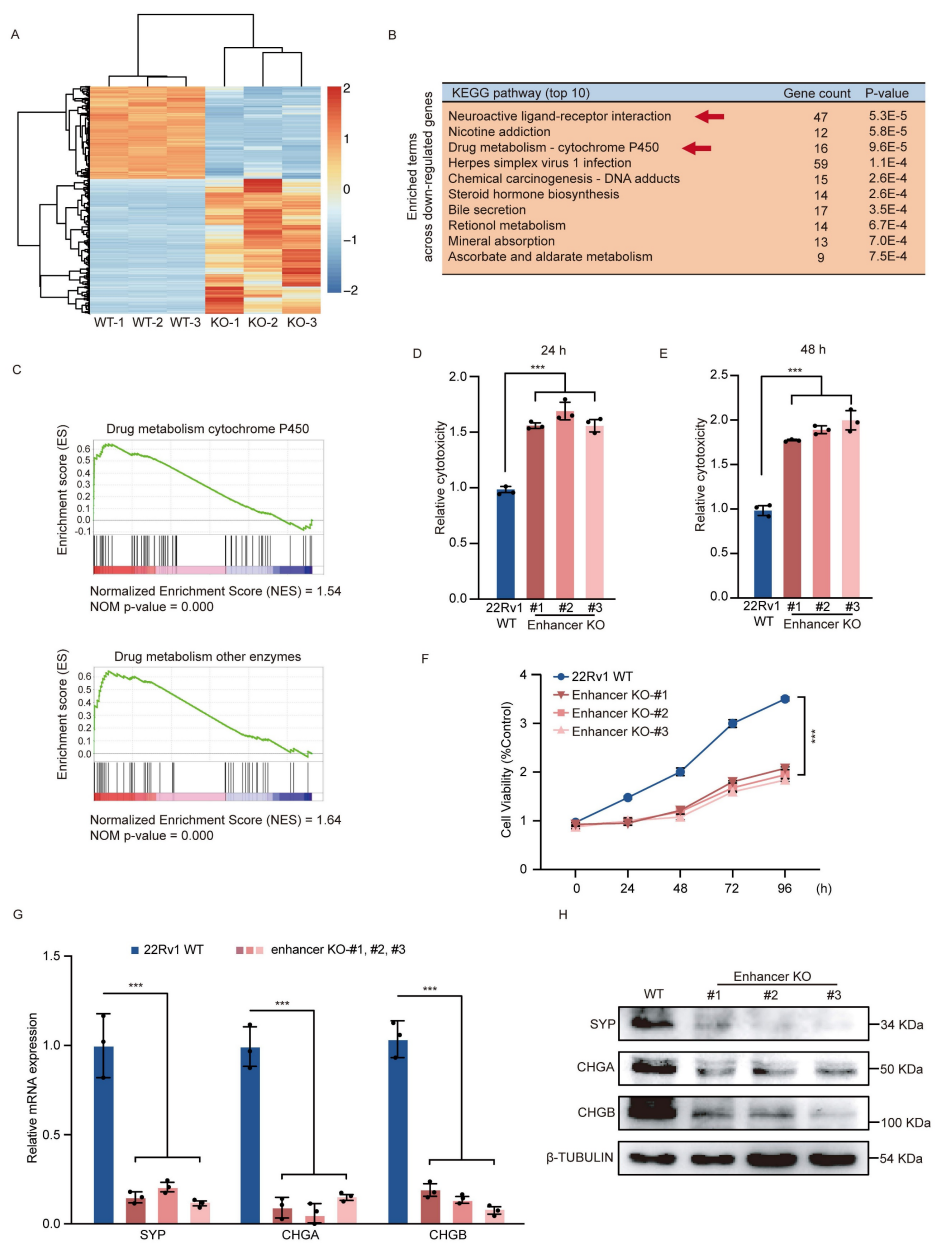
Deletion of rs11067228-related enhancer in PCa cells promotes widespread transcriptomic changes and decreases in malignant phenotypes. (A) Heatmap of differentially-expressed genes in 3 different WT and enhancer KO 22Rv1 lines based on RNA-seq (|log2fold-change| >1.3; p-adjusted value <0.05). (B) KEGG pathway analysis showing biological processes associated with downregulated genes in KO relative to WT cells (Top 10). (C) Gene Set Enrichment Analysis (GSEA) of genes differentially expressed in enhancer KO versus WT 22Rv1 cells (|NES| >1 and NOM p-val <0.05). (Upper) Genes associated with drug metabolism cytochrome p450. (Lower) Genes associated with drug metabolism other enzymes. (D, E) Analysis of LDH release in enzalutamide-treated 22Rv1 WT cells and three similarly-treated enhancer KO lines, as an indicator of cytotoxicity. Release was assayed 24 (D) and 48 (E) hours after treatment. Data represent means ± S.E.M. of three independent experiments. ***P < 0.001. (F) MTS assay in enzalutamide-treated 22Rv1 WT cells and three similarly-treated enhancer KO lines, as an indicator of cell viability. Data represent means ± S.E.M. of three independent experiments. ***P < 0.001. (G) Real-time qPCR validation of transcript levels of indicated NE-related genes (*SYP*, *CHGA* and *CHGB*) in WT 22Rv1 and three enhancer KO lines. Data represent means ± S.E.M. of three independent experiments. ***P < 0.001. (H) Western blot showing levels of NE-related proteins expression in 22Rv1 WT and three enhancer KO lines.

To pinpoint crucial functions of the rs11067228-associated enhancer involved in PCa, we first performed RNA-seq in 22Rv1 WT and enhancer KO cells, using three biological replicates of each. The expression profiles of KO and WT samples are well segregated, as shown in the principal components analysis (PCA) (Fig. S3A). RNA-seq analysis revealed widespread changes in gene expression in KO relative to control WT cells (Fig. 2A). KEGG pathway analysis revealed that down-regulated genes were significantly enriched in neuroactive ligand-receptor interaction and drug metabolism (Fig. 2B). Gene set enrichment analysis (GSEA) also showed that significantly down-regulated genes were enriched in functions related to drug metabolism (Fig. 2C).

To assess the function of risk enhancer in PCa drug sensitivity, we then treated 22Rv1 KO and WT lines with high doses of enzalutamide, based on assessment of lactate dehydrogenase (LDH) release as an indicator of cell damage (Fig. 2D, E). Relative to WT cells, LDH release in enhancer KO cells was significantly increased by enzalutamide treated at 24 h (Fig. 2D) and 48 h (Fig. 2E). We also evaluated MTS assay as an indicator of cell viability in both 22Rv1 WT and KO cells treated with enzalutamide. Relative to WT control, enhancer KO cells treated with enzalutamide showed significantly decreased cell proliferation ability (Fig. 2F), suggesting that enhancer loss increases PCa cell sensitivity to enzalutamide. Given that some PCa cells exhibit drug resistance, lose dependency on AR signals and exhibit lineage plasticity changes, transitioning from adenocarcinoma to highly aggressive neuroendocrine PCa 3-9, we investigated whether risk enhancer can modulate lineage plasticity transition in PCa cells. To validate this hypothesis, we examined the impact of the risk SNP rs11067228-related enhancer on neuroendocrine differentiation of 22Rv1 PCa cells and found that enhancer deletion effectively decreased expression of neuroendocrine markers such as *SYP*, *CHGA*, and *CHGB* (Fig. 2G, H).

To investigate whether the rs11067228-related enhancer functions similarly in other PCa lines, we performed CRISPR interference assays on this enhancer region in C4-2B and LNCaP cells and then assessed drug sensitivity, migration and colony formation phenotypes, as above. Phenotypic defects in C4-2B and LNCaP cells seen after enhancer knockdown were similar with those observed in 22Rv1 cells (Fig. S2), suggesting that the function of the risk SNP rs11067228-related enhancer is likely comparable in most PCa cells, namely, that its activity increases malignant phenotypes and drug resistance.

### The rs11067228-containing enhancer regulates genes expression through long-range interactions in PCa

**Figure 3:**
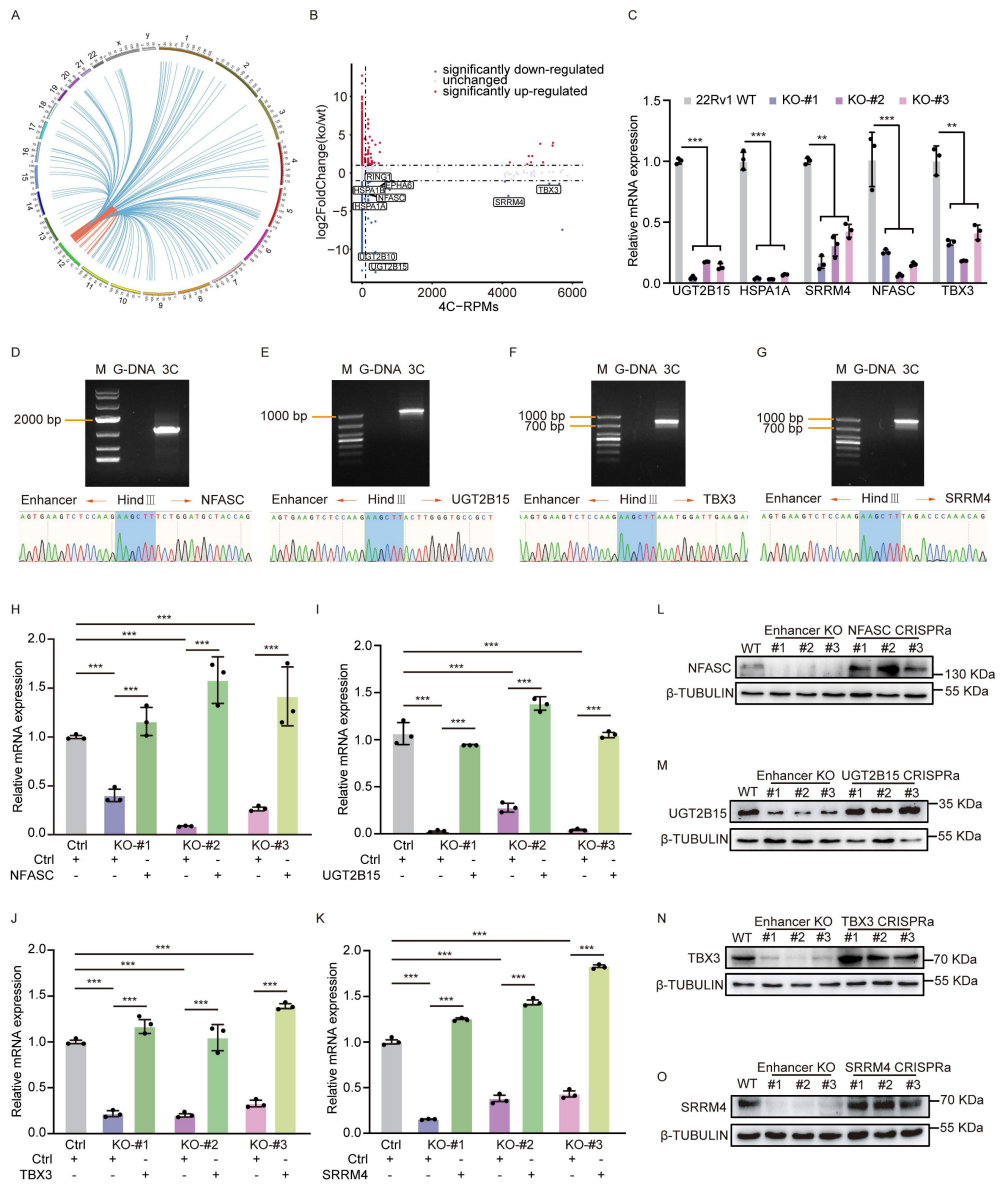
The rs11067228 risk enhancer is a hub for intra- and inter-chromosomal interactions. (A) Circos plot showing genome-wide interactions indicated by curves extending from the enhancer bait locus. For each curve, cis-interactions are depicted in orange and trans-interactions are depicted in blue. Interactions reproducible in two biological replicates are shown. (B) Comparison of fold-changes in gene expression based on RNA-seq and corresponding 4C-Seq signal counts. Boxed genes indicate overlapping down-regulated genes in enhancer KO relative to WT lines with numbers of genome-wide interactions, as indicated by 4C-Seq (log2fold-change < -1.3; p-adjusted value <0.05). (C) Real-time qPCR validation of transcript levels of potential target genes of the risk enhancer based on analysis in enhancer KO cells. Shown are means ± S.E.M. of three independent experiments. ***P < 0.001, **P < 0.01. (D-G) 3C-PCR and sequencing confirmation of interaction of the rs11067228-associated enhancer with loci harboring *NFASC* (D), *UGT2B15* (E), *TBX3* (F) and *SRRM4* (G) genes. Chromatograms confirm respective enhancer and target gene sequences flanking a HindIII linker sequence. M: marker. G-DNA: Genomic DNA, serving as PCR template. 3C: PCR products amplified from the DNA fragments in the 3C library. Forward and reverse PCR primers were designed based on sequences at both enhancer and target gene regions, respectively. (H-O) RT-qPCR and western blot analysis validation of mRNA levels of 4 target genes in WT 22Rv1 cells, 3 enhancer KO lines and enhancer-KO cells subjected to CRISPRa to overexpress indicated target genes individually. Data represents means ± S.E.M. of three independent experiments. ***P < 0.001.

Although some down-regulated genes by enhancer loss could be indirect targets functioning in downstream regulatory networks, we were more interested in direct targets of the rs11067228-related enhancer. Thus, we first identified enhancer contacts by performing circular chromosome conformation capture (4C) analysis in 22Rv1 WT cells, utilizing the noncoding enhancer region containing rs11067228 as the 4C 'bait'. That analysis revealed that 554 sites exerted long-range interactions with the bait region, which spanned across the same or different chromosomes (Fig. 3A and Fig. S3B). Among the 554 interacting sites, 416 (75.09%) are located in cis while 138 (24.91%) are in trans.

Next, we applied a two-step filtering strategy to identify genes directly interacting with the risk enhancer. First, from the RNA-seq data, we selected genes that were significantly down-regulated following enhancer deletion (log2fold-change < -1.3; p-adjusted value < 0.05). Subsequently, we cross-referenced this list with our 4C-seq data, retaining only those genes located within interacting genomic regions that surpassed a stringent signal threshold (RPMs > 50). This process yielded nine high-confidence candidate genes: *UGT2B15*, *UGT2B10*, *HSPA1A*, *HSPA1B*, *EPHA6*, *NFASC*, *TBX3*, *RING1*, and *SRRM4* (Fig. 3B). Some of these genes have been previously linked with cancer progression 48-52. Subsequent validation using quantitative real-time PCR (qRT-PCR) confirmed a significant decrease in expression of a subset 5 candidates (*UGT2B15*, *HSPA1A*, *SRRM4*, *NFASC*, and *TBX3*) following risk enhancer deletion (Fig. 3C). Notably, *TBX3* and *SRRM4* are located on chromosome 12, approximately 13 Kb and 4.3 Mb away, respectively, from the risk SNP; by contrast, *UGT2B15* is located on chromosome 4, *NFASC* on chromosome 1 and *HSPA1A* on chromosome 6. We next performed independent chromosome conformation capture (3C) PCR assays that verified intrachromosomal interactions between the rs11067228-related enhancer and *TBX3* or *SRRM4*, as well as interchromosomal interactions between the enhancer and *UGT2B15* or *NFASC* (Fig. 3D-G and Fig. S3C)*.* However, no significant interaction was detected between the enhancer and the* HSPA1A* locus. Further validation by 3C-qPCR confirmed a specific interaction between the enhancer and the promoters of target genes, as the signal was significantly stronger at the promoters compared to adjacent upstream and downstream regions (Fig. S3D). These results indicated linkage between the risk enhancer and 4 target gene candidates and strongly suggest that the enhancer containing rs11067228 establishes long-range interactions with multiple target genes that could potentially constitute a downstream regulatory network influencing PCa progression.

### A long-range interaction between the risk enhancer and *SRRM4* is associated with enzalutamide sensitivity and neuroendocrine differentiation

**Figure 4:**
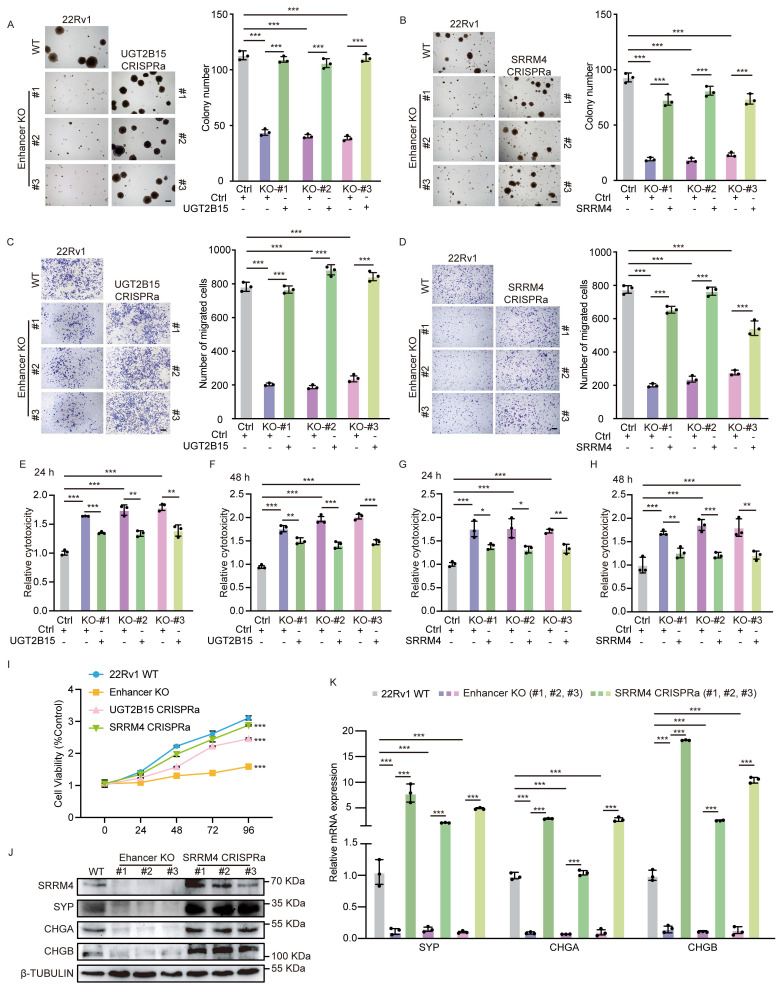
*UGT2B15* and *SRRM4* are essential for the development and maintenance of neuroendocrine differentiation of PCa cells. (A, B) Results of soft agar colony formation assays of WT 22Rv1 cells and three lines each of enhancer KO lines, plus enhancer KO cells subjected to CRISPRa to re-express either *UGT2B15* (A) or *SRRM4* (B) (left). Scale bar =100 μm. Quantification is at right. Data represents means ± S.E.M. of three independent experiments. ***P < 0.001. (C, D) Transwell assays of cells described in (A, B) (left). Scale bar =100 μm. Cells migrated to lower chambers were stained with 0.1% crystal violet. Quantification is at right. Data represents means ± S.E.M. of three independent experiments. ***P < 0.001. (E-H) LDH assays in enzalutamide-treated cells corresponding to those described in (A, B). LDH release was assayed after 24 (E, G) and 48 (F, H) hours of treatment. Data represent means ± S.E.M. of three independent experiments. ***P < 0.001, **P < 0.01. (I) MTS assays in enzalutamide-treated cells corresponding to those described in (A, B). Data represent means ± S.E.M. of three independent experiments. ***P < 0.001. (J) Western blot showing NE-related protein (*SYP*, *CHGA* and *CHGB*) expression in wild-type 22Rv1 cells, as well as three lines each of enhancer-deleted lines plus enhancer-deleted cells re-expressing (by CRISPRa) *SRRM4*. (K) Real-time qPCR validation of transcript levels of NE-related genes in cells corresponding to those described in J. Data represent means ± S.E.M. of three independent experiments. ***P < 0.001.

The results of the above phenotypic experiments showed a positive correlation between risk SNP rs11067228 and NEPC progression. Due to its critical role as an enhancer in drug resistance and neuroendocrine differentiation, rs11067228 exerts long-range chromatin interactions with 4 candidate genes identified above, including *UGT2B15*, *SRRM4*, *NFASC*, and *TBX3.* To determine whether the 4 candidate genes were responsible for observed PCa cell phenotypes, we conducted CRISPR activation assays to individually overexpress each of these genes separately in enhancer KO cells, an activity validated by qRT-PCR and western blot analysis (Fig. 3H-O). Subsequent soft agar colony formation assays demonstrated that re-expression of either *UGT2B15*, *NFASC*, *TBX3*, or *SRRM4* significantly rescued decreased colony formation capacity seen in enhancer-deleted cells (Fig. 4A, B and Fig. S4A, B). Moreover, transwell assays showed that individual re-expression of either *UGT2B15*, *NFASC*, *TBX3*, or *SRRM4* effectively rescued defects in invasive migration seen in enhancer-deleted KO cells (Fig. 4C, D and Fig. S4C, D). No significant change in LDH release was observed in enzalutamide-treated cells overexpressing* NFASC* or* TBX3* relative to enzalutamide-treated enhancer KO cells (Fig.S4E-H). However, over-expression of *UGT2B15* or *SRRM4* partially increased enzalutamide resistance in PCa cells (Fig. 4E-H). MTS cell viability assay further demonstrated that over-expression of *UGT2B15* or *SRRM4* restored the proliferative capacity of PCa cells upon enzalutamide treatment (Fig. 4I), suggesting functional roles of *UGT2B15* and *SRRM4* in CRPC. Consistently, over-expression of *SRRM4* effectively rescued expression of neuroendocrine features in enhancer KO cells, while overexpression of *UGT2B15*, *NFASC* or *TBX3* did not (Fig. 4K and Fig. S4I-K). PCa cell neuroendocrine differentiation was validated by qRT-PCR, western blot analysis and immunofluorescence staining (Fig. 4J, K and Fig.S4L). Functionally, *UGT2B15* encodes an enzyme of the glycosyltransferase superfamily functioning primarily in drug II-phase metabolism and in elimination of toxic compounds 53. *SRRM4* encodes a splicing factor that promoting alternative splicing of neuron-specific exons in target mRNAs. RNA splicing is widely dysregulated in cancer, and *SRRM4* reportedly drives progression of neuroendocrine PCa (NEPC) 52 (Fig. S8C).

**Figure 5:**
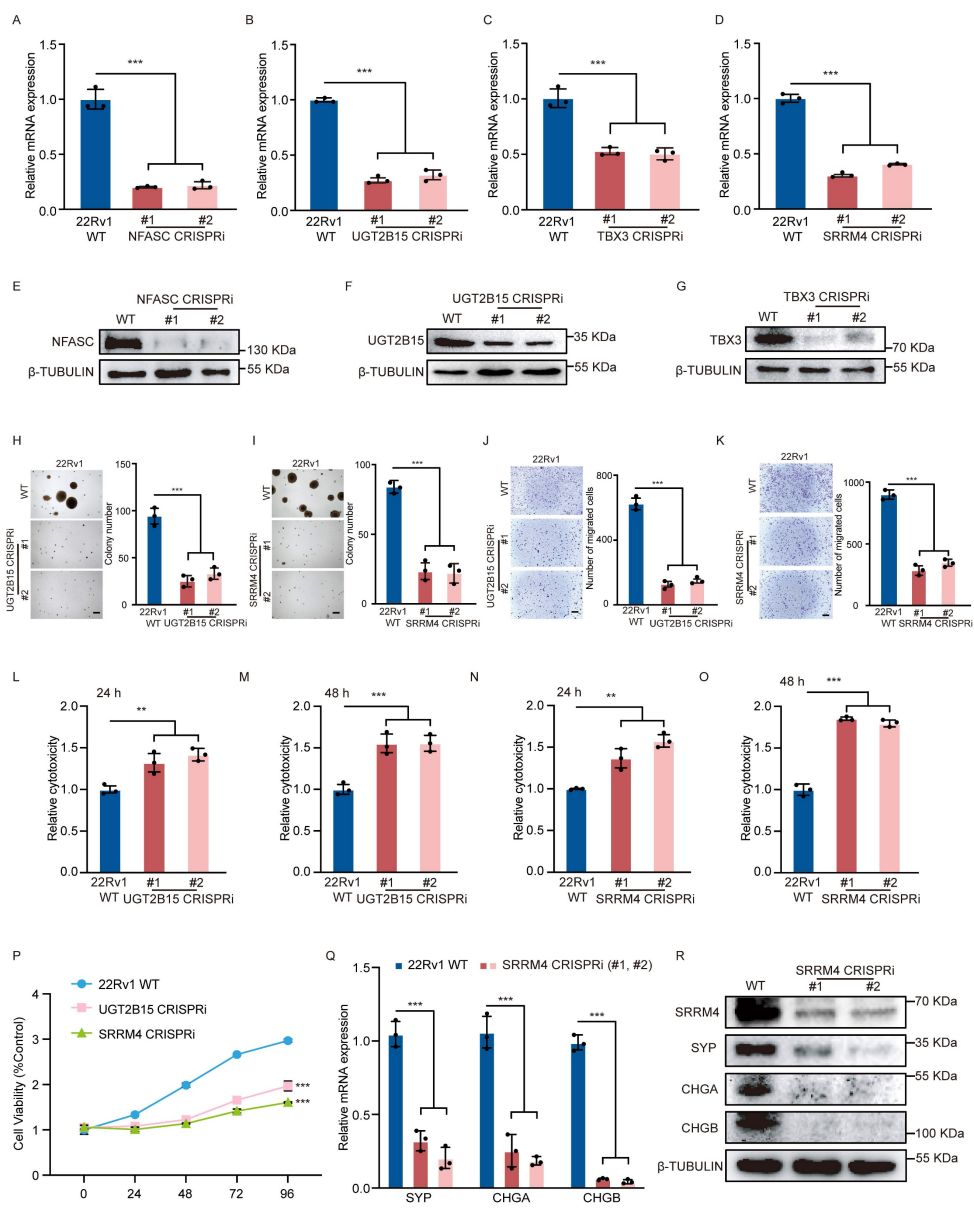
*SRRM4* knockdown blocks PCa cell neuroendocrine differentiation. (A-G, R) Validation of target gene knockdown in PCa cells. Expression of indicated target genes, as measured by real-time qPCR and western blot analysis. sgRNAs targeting gene promoter regions were used in CRISPR-interference (CRISPRi) assays. Data represents means ± S.E.M. of three independent experiments. ***P < 0.001. (H, I) Soft agar colony formation assays in WT control 22Rv1 cells and in cells made deficient in indicated target genes using CRISPRi (left). Scale bar =100 μm. Quantification is at right. Data represents means ± S.E.M. of three independent experiments. ***P < 0.001. (J, K) Transwell assays of cells described in (H, I) (left). Scale bar =100 μm. Cells that had migrated to lower chambers were stained with 0.1% crystal violet. Quantification is at right. Data represents means ± S.E.M. of three independent experiments. ***P < 0.001. (L-O) LDH assays of enzlutamide-treated cells described in (H, I). Assays were performed after 24 (L, N) and 48 (M, O) hours of treatment. Data represent means ± S.E.M. of three independent experiments. ***P < 0.001, **P < 0.01. (P) MTS assays of enzlutamide-treated cells described in (H, I). Data represent means ± S.E.M. of three independent experiments. ***P < 0.001. (Q) Real-time qPCR validation of transcript levels of indicated NE-related genes (*SYP*, *CHGA* and *CHGB*) in cells made deficient in *SRRM4* using CRISPRi. Data represent means ± SEM of three independent experiments. ***P < 0.001. (R) Western blot showing NE-related protein expression in cells made deficient in *SRRM4* using CRISPRi.

Given that re-expression of the 4 target genes (*UGT2B15*, *NFASC*, *TBX3* and *SRRM4)* rescued different malignant phenotypes in PCa cells, we asked how each genes function in PCa. To do so, we conducted CRISPR interference assays to knockdown each gene in 22Rv1 WT cells. qRT-PCR and western blot analysis confirmed gene knockdown (Fig. 5A-G, R). Subsequent soft agar assays demonstrated significantly decreased tumor cell colony formation capacity after knockdown of each of the 4 candidates individually (Fig. 5H, I and Fig. S5A, B). Transwell assays also indicated considerably reduced invasive migration capacity in tumor cells following knockdown of any one of the 4 candidates (Fig. 5J, K and Fig. S5C, D). We also observed significant changes in LDH release (Fig. 5L-O) and cell viability (Fig. 5P) in enzalutamide-treated *UGT2B15* or *SRRM4* knockdown cells relative to enzalutamide-treated WT cells, changes not seen in* NFASC* or *TBX3* knockdown cells (Fig. S5E-H). Furthermore, knockdown of *SRRM4* (Fig. 5Q, R) but not *NFASC*, *UGT2B15* or* TBX3* (Fig. S5I-K) markedly suppressed neuroendocrine differentiation of 22Rv1 cells based on decreased expression of neuroendocrine markers. Collectively, these results suggest that *UGT2B15* may be responsible for drug resistance and that *SRRM4* also functions in drug resistance by promoting neuroendocrine differentiation in PCa cells.

### An allele-specific role for rs11067228 in NEPC

**Figure 6:**
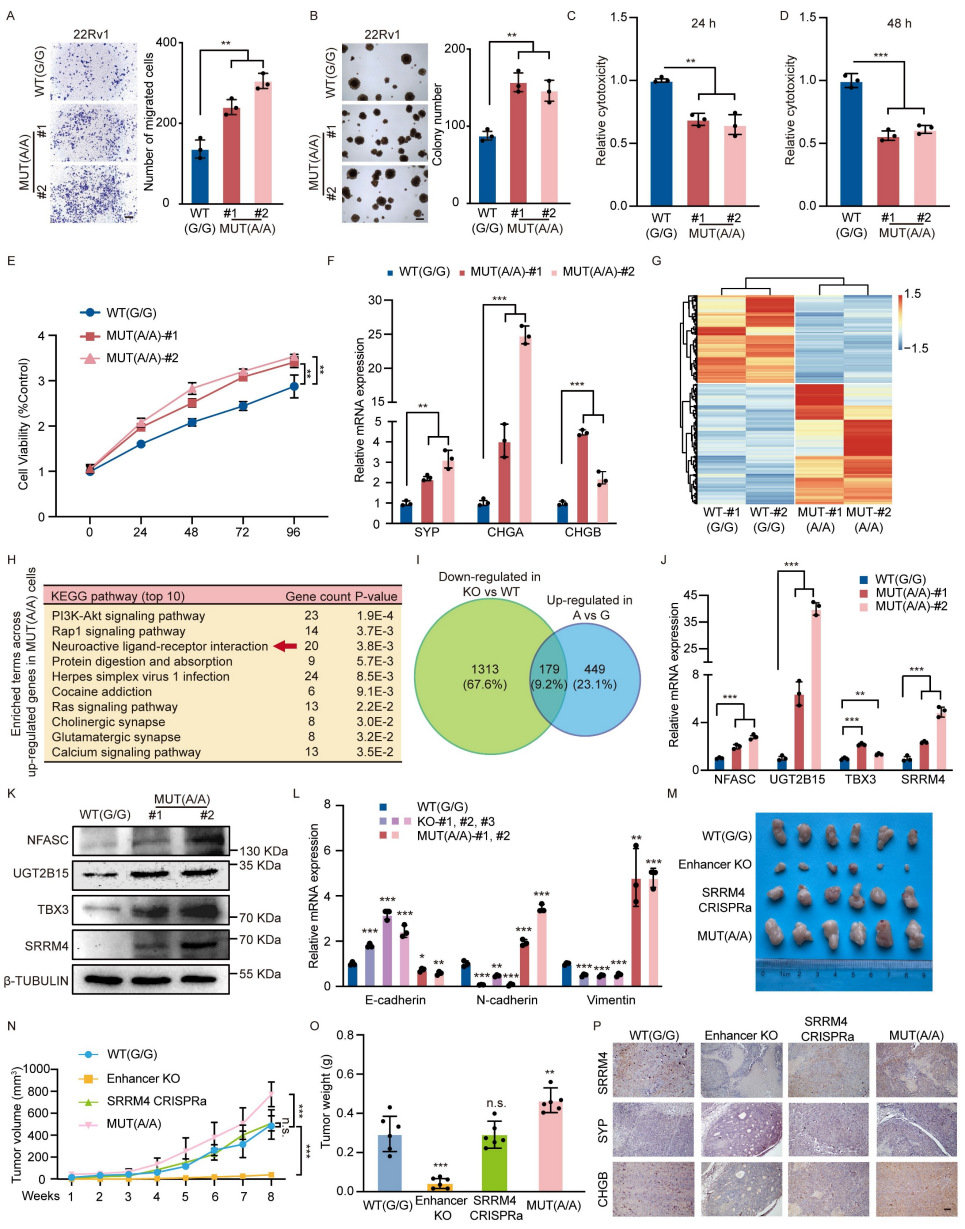
SNP rs11067228 is a functional SNP in 22Rv1 cells. (A) Transwell assays in 22Rv1 cells homozygous for the non-risk (G/G, WT) versus risk (A/A, MUT) alleles of rs11067228. Scale bar =100 μm. Cells that had migrated to lower chambers were stained with 0.1% crystal violet. Quantification is at right. Data represent means ± SEM of three independent experiments. **P < 0.01. (B) Soft agar colony formation analysis of WT (G/G) versus MUT (A/A) 22Rv1 cells. Scale bar =100 μm. Quantification is at right. Data represent means ± SEM of three independent experiments. **P < 0.01. (C, D) LDH assays comparing WT (G/G) and MUT (A/A) cells treated with enzalutamide. LDH release was assayed at 24 (C) and 48 (D) hours after treatment. Data represent means ± S.E.M. of three independent experiments. ***P < 0.001, **P < 0.01. (E) MTS assays comparing WT (G/G) and MUT (A/A) cells treated with enzalutamide. Data represent means ± S.E.M. of three independent experiments. **P < 0.01. (F) Real-time qPCR validation of transcript levels of indicated NE-related genes in WT (G/G) versus MUT (A/A) cells. Data represent means ± SEM of three independent experiments. ***P < 0.001, **P < 0.01. (G) Heatmap of differentially-expressed genes in two samples of 22Rv1 cells harboring the non-risk (G) allele (WT) and two groups of MUT cells harboring the risk (A) allele, based on RNA-seq (|log2fold change| >1 and p-adjusted value <0.05). (H) KEGG pathway showing biological processes of genes upregulated in MUT relative to WT cells (Top 10). (I) Overlap of genes down-regulated in enhancer KO versus WT lines (p-adjusted value <0.05) (green), with the number of genes up-regulated in lines harboring MUT (A/A) versus WT (G/G) cells (log2fold-change >1; p-adjusted value <0.05) (blue). (J, K) Real-time qPCR and western blot analysis validation of transcript levels of indicated target genes of the risk enhancer in MUT and WT cells. Data represents means ± S.E.M. of three independent experiments. ***P < 0.001, **P < 0.01. (L) Real-time qPCR validation of transcript levels of EMT-related genes in WT (G/G), enhancer KO, and MUT (A/A) cells. Data represent means ± SEM of three independent experiments. ***P < 0.001, **P < 0.01, *P < 0.05. (M-O) Photograph showing 22Rv1 xenografts in nude mice with WT (G/G), enhancer KO, enhancer KO cells subjected to CRISPRa to re-express *SRRM4* and MUT (A/A). Tumor volume was measured once a week at indicated time points. Tumor weight was measured when sacrificed (n = 6 per group). Data represent means ± SEM of three independent experiments. ***P < 0.001, **P < 0.01, n.s., not significant. (P) Representative immunohistochemistry staining of indicated 22Rv1 xenografts in (M). Scale bar =100 μm.

As noted above, deletion of the rs11067228-related enhancer down-regulated expression of *UGT2B15* and *SRRM4*, and decreased tumor cell colony formation, invasive migration, drug resistance and neuroendocrine differentiation. To investigate potential differential activities of SNP rs11067228 A and G alleles, we employed CRISPR-Cas9 to perform site-directed mutation of that SNP from G to A in 22Rv1 WT cells (Fig. S6A, B). Sequencing of the approximately 2-kb region encompassing the SNP site confirmed the absence of additional mutations. Transwell assays of resultant clones indicated significantly increased invasive migration of 22Rv1 cells carrying the risk (A) relative to the non-risk (G) allele of rs11067228 (Fig. 6A). Moreover, in soft agar assays, 22Rv1 cells with the risk (A) allele exhibited a ~1.5-fold increase in colony number relative to WT 22Rv1 cells, which harbor the (G) non-risk allele (Fig. 6B). Notably, enzalutamide treatment of 22Rv1 cells harboring the risk (A) allele increased drug resistance and upregulated neuroendocrine markers expression (Fig. 6C-F).

Accordingly, we performed RNA-seq analysis in 22Rv1 cells carrying the risk (A) allele of rs11067228 compared with 22Rv1 WT cells harboring the non-risk (G) allele. This analysis revealed widespread differences in gene expression patterns in the 2 lines (Fig. 6G). KEGG pathway analysis showed that the functions of differentially-expressed genes were primarily related to neuroactive ligand-receptor interactions (Fig. 6H). These expression profiles were consistent with phenotypic changes seen in mutant cells. Among identified gene sets, 1492 genes were down-regulated (P < 0.05) in enhancer KO relative to WT lines, while 628 genes were up-regulated either directly or indirectly (log2fold-change >1; P< 0.05) in cells harboring the risk A allele relative to the non-risk G allele (Fig. 6I). This finding was in contrast to the 1492 genes whose expression was altered by enhancer deletion, indicating that effects of the risk allele are not as global as deletion of the entire enhancer. Strikingly, expression of 179 genes overlapped between both gene sets (Fig. 6I) and the genes were also significantly related to neuroactive ligand-receptor interaction through KEGG pathway analysis (Fig. S6C).

We next sought to validate our four pre-defined target candidates (*NFASC*, *UGT2B15*, *TBX3*, and *SRRM4*). We confirmed that their expression was up-regulated in 22Rv1 cells harboring the rs11067228 risk (A) allele using qRT-PCR and western blot analysis (Fig. 6J, K). Consistently, these genes were also identified within the overlapping gene set that is down-regulated in enhancer-KO cells and up-regulated in A/A versus G/G cells, further corroborating their functional relevance. The observed allele-specific expression patterns prompted us to investigate the underlying mechanism. We hypothesized that the differential gene expression might be driven by variations in chromatin architecture. To test this, we performed 3C-qPCR analysis to measure the physical interaction frequencies between the rs11067228 enhancer and its target promoters in the same isogenic cell models. Strikingly, the interaction frequencies mirrored the expression patterns, with significantly stronger enhancer-promoter looping in MUT(A/A) cells (Fig. S6D).

Having established that the rs11067228 promotes PCa neuroendocrine differentiation, we sought to determine whether it orchestrates a broader spectrum of cellular plasticity. Given the established links between lineage transition, invasiveness, and therapeutic resistance, we evaluated its role in regulating epithelial-mesenchymal transition (EMT) and stemness pathways. Deletion of the rs11067228-related enhancer significantly increased the expression of the epithelial marker *E-cadherin*, while decreasing the mesenchymal markers *Vimentin* and *N-cadherin*, indicative of a shift toward a more epithelial state. Conversely, these mesenchymal markers were upregulated in cells harboring the risk A allele (Fig. 6L).

These above findings support the pivotal role of rs11067228 in driving neuroendocrine differentiation in PCa. To test the functions of rs11067228 in vivo, we subcutaneously transplanted 22Rv1 cells into BALB/c nude mice (n = 6 per group) and found that rs11067228-related enhancer deficiency remarkably reduced both the weight and volume of the xenografts compared with the WT control group while *SRRM4* effectively rescued defects in tumor growth. Furthermore, we showed that 22Rv1 cells carrying the risk (A) of rs11067228 dramatically promoted tumor growth relative to the non-risk (G) allele by measuring the tumor volume and weight of the xenografts (Fig. 6M-O). Immunohistochemistry staining of these tumor sections revealed that risk (A) allele significantly promotes PCa neuroendocrine differentiation and *SRRM4* functionally rescued lineage switching from the neuroendocrine to luminal phenotype caused by rs11067228-related enhancer deficiency (Fig. 6P). The enhanced expression of neuroendocrine markers was accompanied by distinct morphological changes. Histological examination of H&E-stained sections revealed that tumors with the risk A allele or *SRRM4* overexpression exhibited loss of glandular architecture, increased cellular density, and nuclear hyperchromasia, whereas enhancer-knockout tumors retained more differentiated, gland-like structures (Fig. S6E). These observations strongly suggest an allele-specific role for SNP rs11067228 in enhancer function and in NEPC.

To assess the generalizability of our findings, we sought to introduce the risk A allele into another prostate cancer cell line, C4-2B. Due to the polyploid nature of commonly available models, we obtained a heterozygous clone (Fig. S6F). Notably, even in this heterozygous context, we observed a significant shift in target gene *SRRM4* and neuroendocrine markers expression relative to wild-type controls (Fig. S6G). This supports the notion that the rs11067228-A allele exerts a functional impact across different cellular contexts.

### The transcription factor SOX4 preferentially binds to the risk allele to modulate long-range chromatin interaction

**Figure 7:**
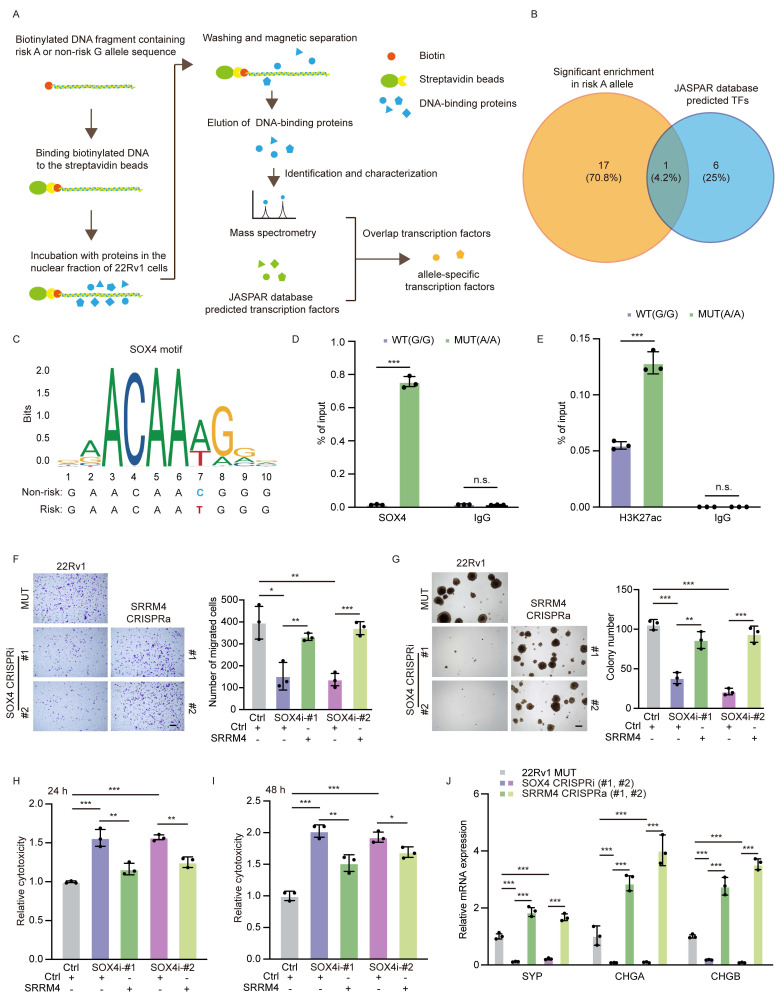
The transcription factor SOX4 preferentially bind to the rs11067228 risk allele to modulate target gene expression. (A) Schematic showing DNA-protein pull-down assays and mass spectrometry (MS) analysis to identify TFs binding to the rs11067228 risk allele. (B) Overlap in the number of TFs significantly enriched at the risk (A) relative to non-risk (G) allele (orange) with numbers of predicted TF motifs analyzed in the JASPAR database (blue). (C) The sequence of a potential SOX4 binding motif differs in the risk (A) versus non-risk (G) alleles of rs11067228. (Upper) Predicted preferential SOX4 binding site, based on the JASPAR database. (Lower) Shown are actual sequences of non-risk and risk alleles. (D) Quantification of ChIP-qPCR analysis showing SOX4 enrichment at the rs11067228 locus in indicated WT (G/G) versus MUT (A/A) cells. Data represents means ± S.E.M. of three independent experiments. ***P < 0.001, n.s., not significant. (E) Quantification of ChIP-qPCR analysis showing H3K27ac enrichment at the rs11067228 locus in indicated WT (G/G) versus MUT (A/A) cells. Data represents means ± S.E.M. of three independent experiments. ***P < 0.001, n.s., not significant. (F) Transwell assays of MUT 22Rv1 cells and two lines each of *SOX4* knockdown lines, plus *SOX4* knockdown cells subjected to CRISPRa to re-express *SRRM4*. Cells migrated to lower chambers were stained with 0.1% crystal violet. Scale bar =100 μm. Quantification is at right. Data represents means ± S.E.M. of three independent experiments. ***P < 0.001, **P < 0.01, *P < 0.05, n.s., not significant. (G) Results of soft agar colony formation assays of cells described in (F) (left). Scale bar =100 μm. Quantification is at right. Data represents means ± S.E.M. of three independent experiments. ***P < 0.001, **P < 0.01. (H, I) LDH assays in enzalutamide-treated cells corresponding to those described in (F). LDH release was assayed after 24 (H) and 48 (I) hours of treatment. Data represent means ± S.E.M. of three independent experiments. ***P < 0.001, **P < 0.01, *P < 0.05. (J) Real-time qPCR validation of transcript levels of NE-related genes (*SYP*, *CHGA* and *CHGB*) in cells described in (F). Data represent means ± S.E.M. of three independent experiments. ***P < 0.001.

Differential TF binding can alter expression of genes subject to SNP-dependent cis- or trans-regulation. Given its allele-specific function, we next asked what TFs bind to the risk rs11067228 sequence by employing JASPAR, an open-access database of TF binding profiles, to identify factors binding to that site. Concurrently, we used DNA-protein pull-down assays followed by mass spectrometry (MS) analysis to identify DNA-protein interactions (Fig. 7A). Then, after filtering out low- or non-expressed proteins in 22Rv1 cells, we focused on 7 allele-specific TFs (Fig. S7A). We then compared proteins bound to DNA probes representing the 2 SNP rs11067228 alleles, with a focus on proteins preferentially binding to the risk (A) versus the non-risk (G) allele. Notably, 18 TFs exhibited significant enrichment in risk (A) relative to non-risk (G) alleles. Among these, SOX4 protein overlapped between the JASPAR database and our DNA-protein pull-down assays, making it a primary candidate (Fig. 7B). Motif analysis based on the JASPAR database showed that rs11067228 overlaps with a SOX4 binding motif (Fig. 7C). To confirm these predictions, we conducted chromatin immunoprecipitation with a SOX4 antibody followed by quantitative PCR (ChIP-qPCR). That analysis indicated that SOX4 exhibited a higher preference for the risk allele “A” versus the non-risk allele “G” allele (Fig. 7D). Notably, the risk (A) allele exhibited a ~8-fold enrichment in the SOX4 binding relative to the non-risk (G) allele (Fig. S7B).

Having established that SOX4 preferentially binds the risk A allele, we next asked whether this differential transcription factor occupancy translates into differences in enhancer activity. To this end, we performed H3K27ac ChIP-qPCR in A/A versus G/G cell lines. The analysis revealed that the pre-existing H3K27ac mark at this enhancer was significantly augmented by the presence of the risk A allele (Figure 7E). This allele-specific epigenetic modification suggests that the preferential binding of SOX4 to the A allele fosters a more open chromatin configuration, functionally poising the enhancer for stronger transcriptional activity.

To further validate the allele-specific functional role of SOX4 in high-order chromatin interactions, we performed gain- and loss-of-function experiments in MUT(A/A) and WT(G/G) cells. This analysis revealed that SOX4's regulatory impact was significantly potentiated in the presence of the risk A allele. SOX4 overexpression induced a substantially stronger upregulation of target genes in A/A cells compared to G/G cells (Fig. S7E, F). Similarly, SOX4 knockdown resulted in a more profound suppression of target genes in A/A cells (Fig. S7G, H).

Having established that SOX4 exhibits genotype-dependent regulation of multiple target genes, we next sought to identify the critical downstream effector responsible for PCa progression. To this end, we employed a functional rescue approach in which we individually re-expressed each candidate target gene in the SOX4 knockdown cells. Notably, knockdown of *SOX4* expression strongly reduced tumor cell colony formation, invasive migration, drug resistance and neuroendocrine differentiation, while over-expression of *SRRM4* effectively rescued above deficiencies in *SOX4* knockdown cells (Fig. 7F-J).

We next asked whether SOX4's regulatory function depends specifically on the rs11067228-related enhancer. Using CRISPRa to overexpress *SOX4* across three different cell lines, we found that *SOX4*-mediated upregulation of *SRRM4* was significantly blunted in KO cells, demonstrating the essential requirement of this enhancer for SOX4 function (Fig. S7I). Strikingly, in cells harboring the risk A allele, *SOX4* overexpression resulted in enhanced *SRRM4* upregulation compared to WT cells, indicating that the A allele potentiates SOX4's transcriptional efficacy. Collectively, our data delineate a coherent mechanistic pathway: the risk A allele of the rs11067228 creates a high-affinity binding site for TF SOX4, which in turn recruits epigenetic writers to amplify the pre-existing H3K27ac mark, thereby enhancing the interaction between risk enhancer and *SRRM4* to promote castration-resistant and neuroendocrine prostate cancer.

## Discussion

Genetic factors play a critical role in PCa development and progression 54, 55, as well in both PCa resistance to ADT and neuroendocrine differentiation. PCa cell growth is highly dependent on the androgen/ AR signaling axis, and ADT is the foundation of clinical treatment of PCa 1, 2. Under treatment pressure, adenocarcinoma cells undergo multiple genomic alterations and epigenomic reprograming to activate different pathways, often acquiring stemness and undergoing a transformation toward a mesenchymal state. Neuroendocrine prostate cancer (NEPC) is a highly malignant PCa subtype with low androgen dependence and aggressive metastasis. So far, there are no effective treatment options for NEPC, and the prognosis is extremely poor 3-9. GWAS identified SNP rs11067228 as significantly associated with castration-resistant metastasis (CM) 27. In our study, we found that the region containing SNP rs11067228 is a PCa-specific active enhancer, and that the enhancer is an essential, functional element regulating in PCa drug resistance and neuroendocrine differentiation.

Chromosome conformation capture technologies have enabled studies of the dynamics of high-order chromatin structures and long-range chromatin interactions 30-32, 56. The non-coding variant rs1800734 enhances *DCLK3* expression through long-range interactions and promotes colorectal cancer progression 57. We reported that the *HOTAIR* regulatory element modulates glioma cell sensitivity to temozolomide through long-range regulation of multiple target genes 58. Furthermore, we reported that the PCa risk variant rs55958994 regulates expression of multiple gene through extremely long-range chromatin interactions to control PCa progression 36. However, whether long-range chromatin interactions play a significant role in CRPC and NEPC has not been characterized. Here, 4C assays and RNA-seq performed in 22Rv1 WT and enhancer KO cells revealed that the rs11067228-associated enhancer modulates expression of multiple candidate genes, among them, *UGT2B15*, *NFASC*, *TBX3* and *SRRM4*—two (*TBX3* and *SRRM4*) on the same chromosome as the enhancer and two (*UGT2B15* and *NFASC*) on the other chromosomes—strongly suggesting that long-range intra- and inter-chromosomal chromatin interactions may underlie PCa progression. The quantitative 3C-qPCR analysis provides independent validation of the chromatin interactions initially detected by our 4C-seq. The precise measurement of interaction frequencies and demonstration of spatial specificity firmly establish that the rs11067228 enhancer engages in stable, specific looping with its target promoters.

Interestingly, we found that re-expression of each target gene effectively rescued tumorigenic phenotypes seen in PCa cells lacking the entire enhancer. Specifically, restoring *UGT2B15* or* SRRM4* expression increased enzalutamide sensitivity in enhancer-deleted cells. *UGT2B15* reportedly serves as the major *UGT* enzymes functioning in drug II-phase metabolism and is responsible for local DHT glucuronidation in human prostate cells 48. Changes in androgen glucuronidation reportedly function in PCa progression 59. However, PCa progression is highly heterogeneous, with approximately 30% of metastatic CRPC cases eventually evolving into a neuroendocrine phenotype. Our findings reveal that, in addition to its local effect on *UGT2B15*, rs11067228 engages in long-range chromatin interactions to modulate *SRRM4* expression. RNA splicing is widely dysregulated in cancer, and *SRRM4* alters the sequence of mRNA encoding the *RE1* silencing transcription factor (*REST*) to perturb its activity, promoting PCa neuroendocrine differentiation 60, 61. Moreover, *SRRM4* re-expression rescued decreased expression of neuroendocrine markers seen in enhancer-deleted cells. We propose that rs11067228 functions as a pleiotropic risk variant. While it may support tumor survival via metabolic adaptation (*UGT2B15*) in adenocarcinoma, it specifically facilitates lineage plasticity (*SRRM4*) in the subset of tumors undergoing neuroendocrine trans sdifferentiation. Therefore, our study does not contradict the *UGT2B15* mechanism but rather expands the functional repertoire of rs11067228, identifying it as a critical 'plasticity enabler' that facilitates the transition toward the neuroendocrine phenotype in advanced disease stages.

To define SNP activity it is crucial to validate functional effects of different alleles on gene expression. For example, the functional variant rs34330 of *CDKN1B* is associated with risk of neuroblastoma 62. SNP rs61752561 modulates stability and conformation of PSA protein, and creates an extra-glycosylation site 63. Moreover, SNP rs138213197 located in a *HOXB13* exon represents a recurrent mutation (G84E) 64. Our investigations reveal that mutating SNP rs11067228 from the G to the A allele altered tumor cell colony formation, invasive migration, enzalutamide sensitivity and neuroendocrine differentiation. The allele-specific enhancement of chromatin looping provides the mechanistic basis for the differential gene regulation observed between A/A and G/G cells. The stronger physical interaction between the enhancer and target promoters in A/A cells likely facilitates more efficient recruitment of the transcriptional machinery, thereby amplifying the expression of oncogenic drivers like SRRM4. These findings establish a direct link between genetic variation at rs11067228, three-dimensional genome architecture, and ultimately, prostate cancer progression. Interestingly, while the rs11067228 enhancer robustly induced EMT and neuroendocrine markers, it did not consistently alter the expression of core stemness factors such as *OCT4, SOX2* and *NANOG*. This suggests that the enhancer's pro-metastatic function may be specifically channeled through driving specific lineage plasticity programs rather than a global stem-like state, highlighting the context-dependent nature of tumor cell de-differentiation.

Transcriptional regulation by TFs plays a crucial role in tumor progression. Previous studies report that rs2280381 alleles modulate binding of the TF PU.1 to differentially regulate *IRF8* expression in autoimmune diseases 65. Risk variants of rs11672691 and its LD SNP rs887391 modulate binding of the TFs NKX3.1 and YY1 to the *PCAT19* promoter, resulting in promoter-enhancer switching in PCa 23. Here, we identified SOX4 as allele-specific TF binding to the rs11067228 locus by integrating JASPAR database analysis with DNA-protein pull-down data. Moreover, *SOX4* overexpression significantly activated expression of target genes regulated by the rs11067228-related enhancer. Our allele-specific binding and functional data collectively support a model where the regulatory output of SOX4 is gated by the rs11067228 genotype. The ChIP-qPCR analysis demonstrates that under physiological conditions, SOX4 binds with high affinity and specificity to the risk-associated A allele, but not to the G allele (Fig. 7D). This A-allele-specific occupancy is the molecular event that nucleates the enhancer-promoter interactions necessary for target gene activation. The observation that ectopic overexpression of SOX4 can perturb gene expression even in G-allele cells likely reflects a supraphysiological phenomenon, wherein extremely high transcription factor concentrations can force occupancy at low-affinity sites. This does not diminish the biological relevance of the allele-specific binding but rather underscores it: the A allele confers a high-affinity platform that allows for efficient SOX4 recruitment and pathway activation at native expression levels, thereby lowering the threshold for oncogenic pathway engagement and explaining its genetic association with disease risk (as demonstrated by our functional assays). This model explains why SOX4 manipulation produces effects in both genotypes, but with substantially amplified magnitude in A/A cells. Our functional dissection reveals a hierarchical relationship within the SOX4-regulated gene network. While SOX4 coordinately modulates the expression of several genes in an allele-specific manner, *SRRM4* emerges as the dominant downstream effector responsible for executing SOX4's pro-tumorigenic functions. This finding is consistent with *SRRM4*'s established role as a master regulator of alternative splicing in neuroendocrine differentiation and provides a mechanistic explanation for how the rs11067228-SOX4 axis ultimately drives cancer progression.

In summary, our study reveals that risk SNP rs11067228 is a key genetic variant associated with PCa castration-resistance and neuroendocrine differentiation. This function establishes a link between PCa risk genetic variants and gene expression changes governed by intra- and inter-chromosomal long-range interactions, coupled with preferential binding of allele-specific TFs. This work captures the essence of the post-GWAS research by analyzing complex relationships between risk genetic factors and PCa disease progression. Defining these links should advance our comprehension of intrinsic molecular mechanisms underlying effects of risk SNP rs11067228 on NEPC, define relevant upstream and downstream factors and signaling pathways, and provide experimental support for more effective therapies against NEPC.

## Supplementary Material

Supplementary figures and tables.
