# Supplementary material for: The castration-resistant prostate cancer-associated SNP rs11067228 facilitates neuroendocrine differentiation through an enhancer-mediated chromatin interaction with SRRM4
Source: Int J Biol Sci. 2026 Jan 8;22(3):1440–60. doi: 10.7150/ijbs.124731 (PMC12837743; doi:10.7150/ijbs.124731)
Supplement: Supplementary file 1 — Supplementary figures and tables. [file ijbsv22p1440s1.pdf]

# Supplementary Materials for

## The castration-resistant prostate cancer-associated SNP rs11067228 facilitates neuroendocrine differentiation through an enhancer-mediated chromatin interaction with SRRM4

**Yuan Jiang[1, 3], Zhenhao Zhao[1], Peng Li[1], Guangsong Su[2], Yuyang Qian[1], Yuting Zhao[1], Bo Wang[1], Yunlong Bai[1], Lei Zhang[1], Zhongfang Zhao[1], Jiandang Shi[1,*], and Wange Lu[1, 2, 4*]**

[1] State Key Laboratory of Medicinal Chemical Biology, College of Life Sciences, Nankai University, 300071 Tianjin, People's Republic of China

[2] Department of Laboratory Medicine and Institute of Precise Medicine, The First Affiliated Hospital, Sun Yat-sen University, 510080 Guangzhou, Guangdong, People's Republic of China

[3] Weifang People’s Hospital, Shandong Second Medical University, 261000 Weifang, Shandong, People's Republic of China

[4] Lead contact

* **Correspondence**: Wange Lu: wangelv@gmail.com;

Jiandang Shi: shijd@nankai.edu.cn

**This file includes:**

Fig. S1. CRISPR/Cas9-mediated deletion of the rs11067228-associated enhancer.

Fig. S2. Deletion of the rs11067228-related enhancer decreases

malignant phenotypes in LNCaP and C4-2B cells.

Fig. S3. Integrated analysis of gene expression and chromatin architecture.

Fig. S4. Rescue by candidate target genes of malignant phenotypes seen in 22Rv1 cells after deletion of the rs11067228 risk enhancer.

Fig. S5. Knockdown of target genes partially rescues malignant phenotypes of 22Rv1 PCa cells.

Fig. S6. Functional characterization of the rs11067228 A allele across prostate cancer models.

Fig. S7. Identification and functional validation of SOX4 as the key transcription factor mediating rs11067228 enhancer activity.

Fig. S8. Combined prognostic value of *SRRM4*/*UGT2B15* and their subtype-specific expression patterns in prostate cancer.

Table S1. Guide RNAs and primers used to assess CRISPR/Cas9-mediated deletions.

Table S2. Primers used in the qRT-PCR experiment.

Table S3. Primers used for the 3C-PCR assay.

Table S4. Guide RNAs and primers used to assess CRISPR/Cas9-mediated SNP editing.

Table S5. Primers used to construct the luciferase reporter plasmid.

Table S6. Primers used for 4C analysis.

Table S7. sgRNA sequences used in CRISPRa and CRISPRi assays.

Table S8. Sequences of biotinylated double-strand oligonucleotides used in pull-down assays.

Table S9. 3C-qPCR primer sequences and amplification efficiencies.

Table S10. Key resources table.

## Supplementary Figure 1

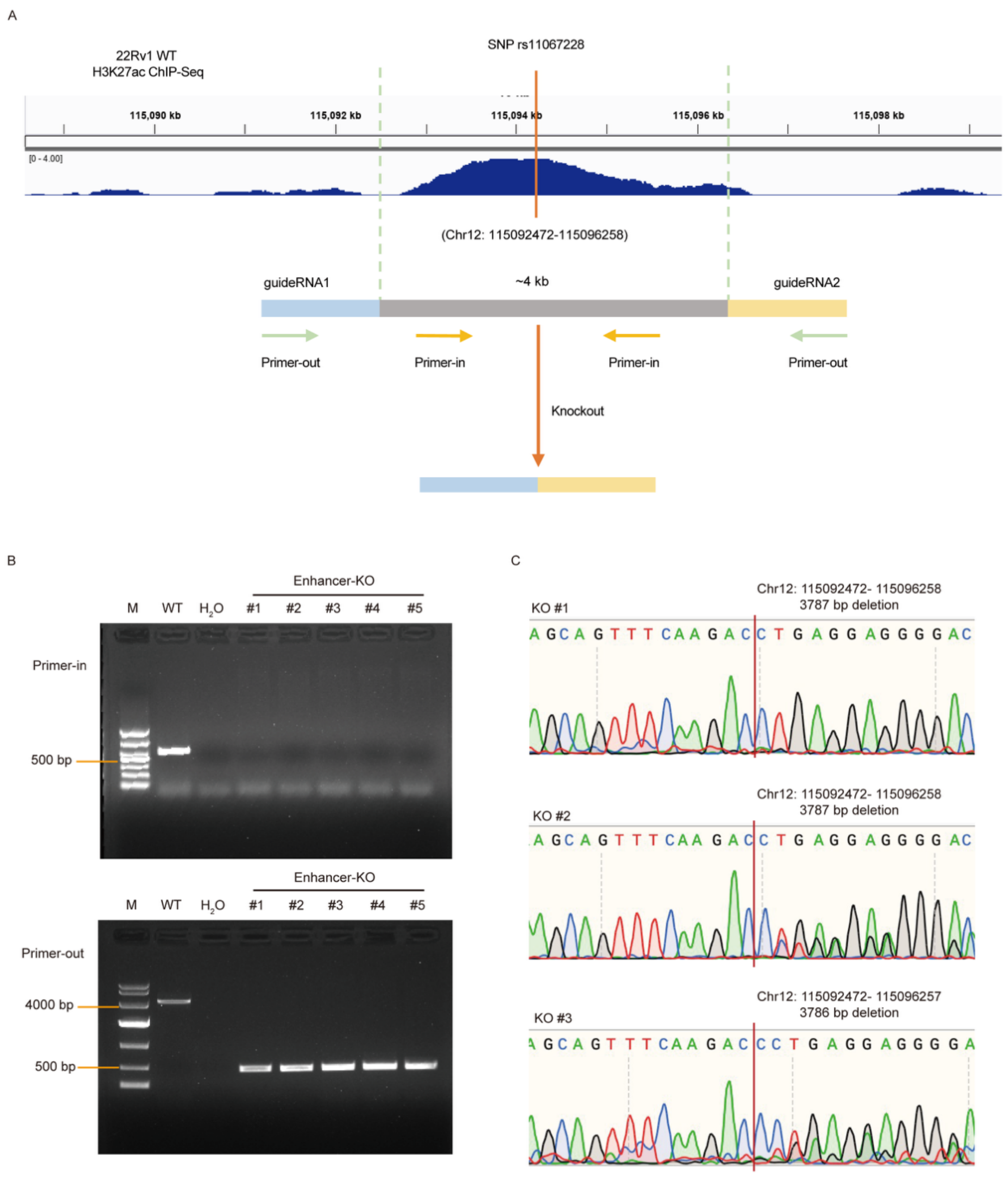


Figure S1. CRISPR/Cas9-mediated deletion of the rs11067228-associated enhancer. (A) Schematic showing the deleted 4 kb region and positions of knockout screening primers. (B) PCR analysis used to identify knockout cell

clones. Note the absence of a PCR product with “primer-in” (upper) and the appearance of 500bp PCR products with “primer-out” (lower), confirming deletion of the enhancer region. M: marker; $H_2O$: Negative control. (C) Sequencing of the deletion breakpoint described in (A) in relevant knockout clones generated by CRISPR/Cas9 editing. KO-#1: 3787 bp deletion (hg19, Chr12:115092472-115096258); KO-#2: 3787 bp deletion (hg19, Chr12:115092472-115096258); KO-#3: 3786 bp deletion (hg19, Chr12:115092472-115096257).

# Supplementary Figure 2

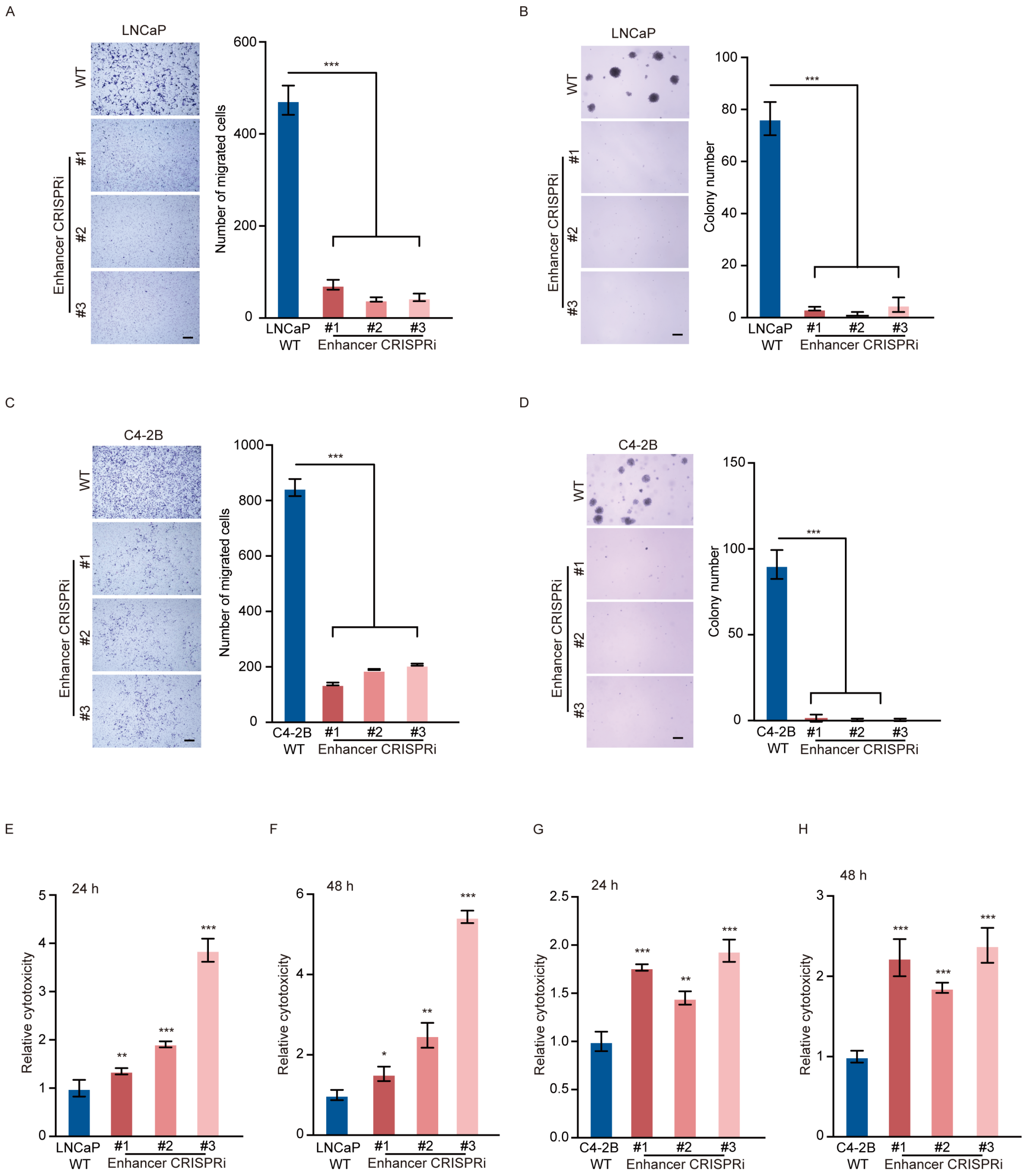


Figure S2. Deletion of the rs11067228-related enhancer decreases malignant phenotypes in LNCaP and C4-2B cells. (A) Transwell assays of LNCaP WT and enhancer-deleted cells generated by CRISPRi. Cells migrating to lower

chambers were stained with 0.1% crystal violet (left). Scale bar =100 μm. Quantification of migrated cells in corresponding cells (right). Data represent means ± S.E.M. of two independent experiments. ***P < 0.001. (B) Analysis of colony formation in soft agar of cells corresponding to those described in A (left). Scale bar =100 μm. Quantification is at right. Data represent means ± S.E.M. of two independent experiments. ***P < 0.001. (C) Transwell assays of C4-2B WT and enhancer-deleted cells generated using CRISPRi. Cells migrating to lower chambers were stained with 0.1% crystal violet (left). Scale bar =100 μm. Quantification of migrated cells is shown at right. Data represent means ± S.E.M. of two independent experiments. ***P < 0.001. (D) Analysis of colony formation in soft agar of cells corresponding to those described in C (left). Scale bar =100 μm. Quantification is at right. Data represent means ± S.E.M. of two independent experiments. ***P < 0.001. (E, F) Results of LDH release assays performed to assess cytotoxicity in cells described in A. After enzalutamide (50 nM) treatment, LDH release was assayed at 24 (E) and 48 (F) h. Data represent means ± S.E.M. of three independent experiments. ***P < 0.001, **P < 0.01, *P < 0.05. (G, H) Results of an LDH release assay in cells described in C. After enzalutamide (80 nM) treatment, LDH release was assayed at 24 (G) and 48 (H) h. Data represent means ± S.E.M. of three independent experiments. ***P < 0.001, **P < 0.01.

# Supplementary Figure 3

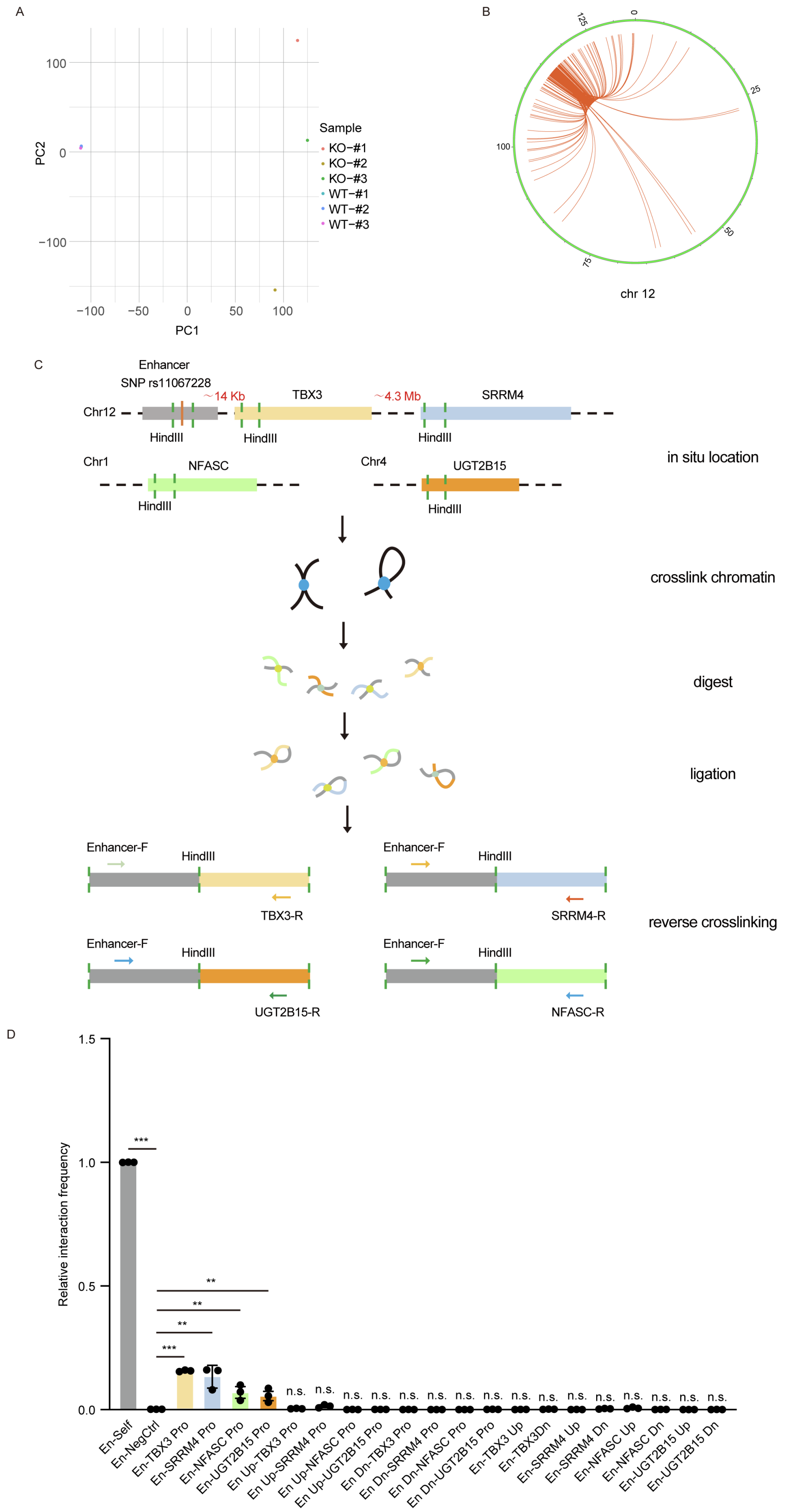

Figure S3. Integrated analysis of gene expression and chromatin architecture. (A) Principal component analysis of RNA-seq results. Triplicates of KO samples and triplicates of WT samples were analyzed. (B) The circus plot visualizing cis-interactions indicated by curves extending from the enhancer bait locus. (C) Schematic representation of the 3C experimental strategy for detecting chromatin interactions between the rs11067228-containing enhancer and its target genes. (D) Quantitative 3C-qPCR analysis of enhancer-promoter interactions. Data represent efficiency-corrected interaction frequencies normalized to anchor self-ligation. Data represent means ± S.E.M. of three independent experiments. ***P < 0.001, **P < 0.01, n.s., not significant.

# Supplementary Figure 4

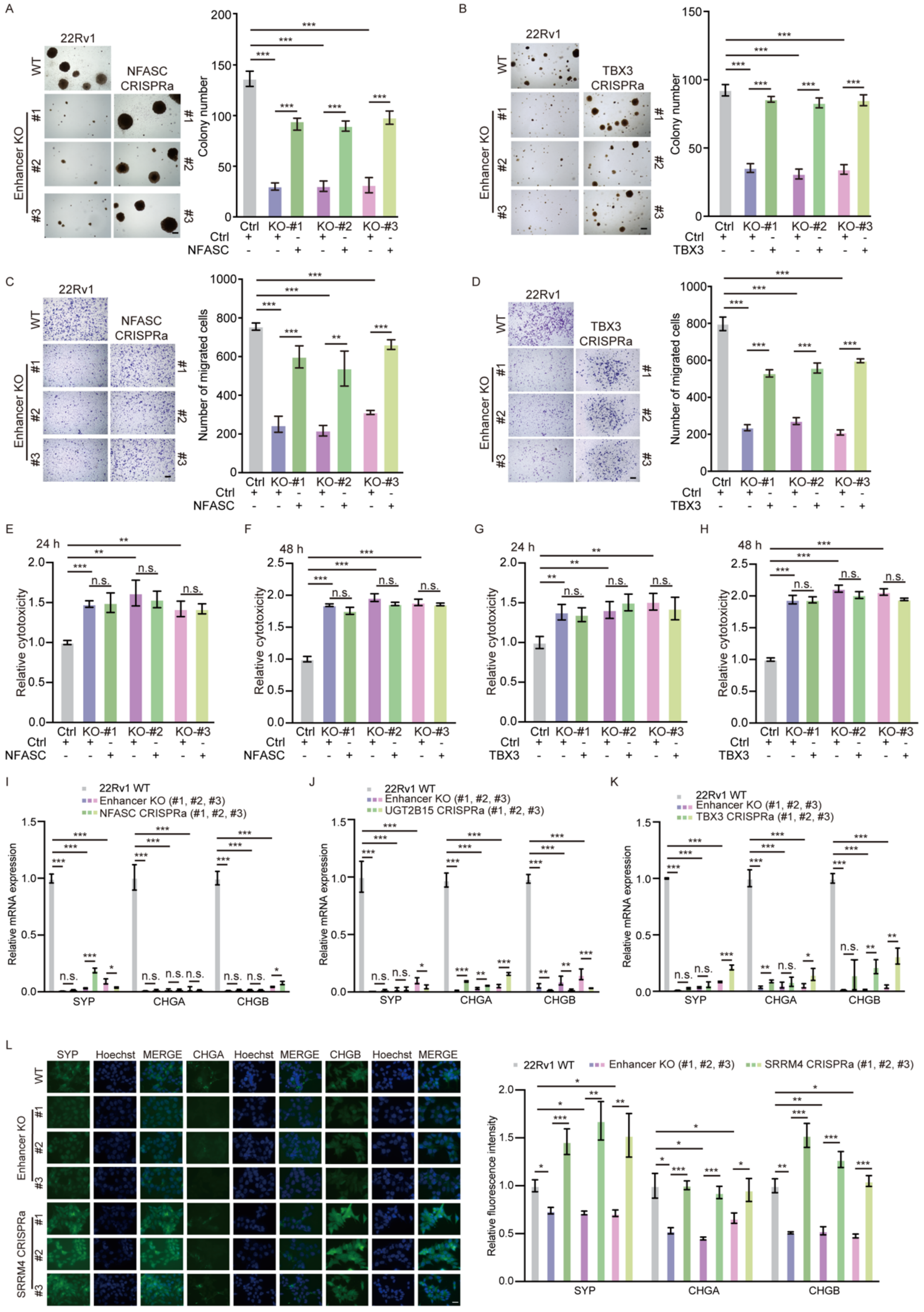


Figure S4. Rescue by candidate target genes of malignant phenotypes seen in

22Rv1 cells after deletion of the rs11067228 risk enhancer. Target genes were overexpressed using CRISPRa. (A, B) Soft agar colony formation assays in WT 22Rv1 cells and in three lines each of enhancer-deleted lines plus 3 enhancer-deleted cells overexpressing the indicated (*NFASC* and *TBX3*) genes (left). Scale bar =100 μm. Quantification is at right. Data represents means ± S.E.M. of three independent experiments. ***P < 0.001. (C, D) Transwell assays of enhancer-deleted and corresponding rescued cells described above (left). Scale bar =100 μm. Cells migrated to lower chambers were stained with 0.1% crystal violet. Quantification is at right. Data represents means ± S.E.M. of three independent experiments. ***P < 0.001, **P < 0.01. (E-H) LDH assays in cells corresponding to those described above and treated with enzalutamide (100 μM) at 24 (E, G) and 48 (F, H) h. Data represent means ± S.E.M. of three independent experiments. ***P < 0.001, **P < 0.01, n.s., not significant. (I-K) Real-time qPCR validation of transcript levels of NE-related genes (*SYP*, *CHGA* and *CHGB*) in WT 22Rv1 cells plus 3 enhancer-deleted cells overexpressing the indicated (*NFASC*, *UGT2B15* and *TBX3*) genes. Data represent means ± S.E.M. of three independent experiments. ***P < 0.001, **P < 0.01, *P < 0.05, n.s., not significant. (L) Representative immunofluorescence staining of NE-related genes (*SYP*, *CHGA* and *CHGB*) in wild-type 22Rv1 cells, as well as three lines each of enhancer-deleted lines plus enhancer-deleted cells re-expressing (by CRISPRa) *SRRM4* (left). Scale bar =10 μm. Relative fluorescence intensities were quantified in ~100 nuclei for each condition and

plotted (right). ***P<0.001， **P<0.01， *P<0.05.

# Supplementary Figure 5

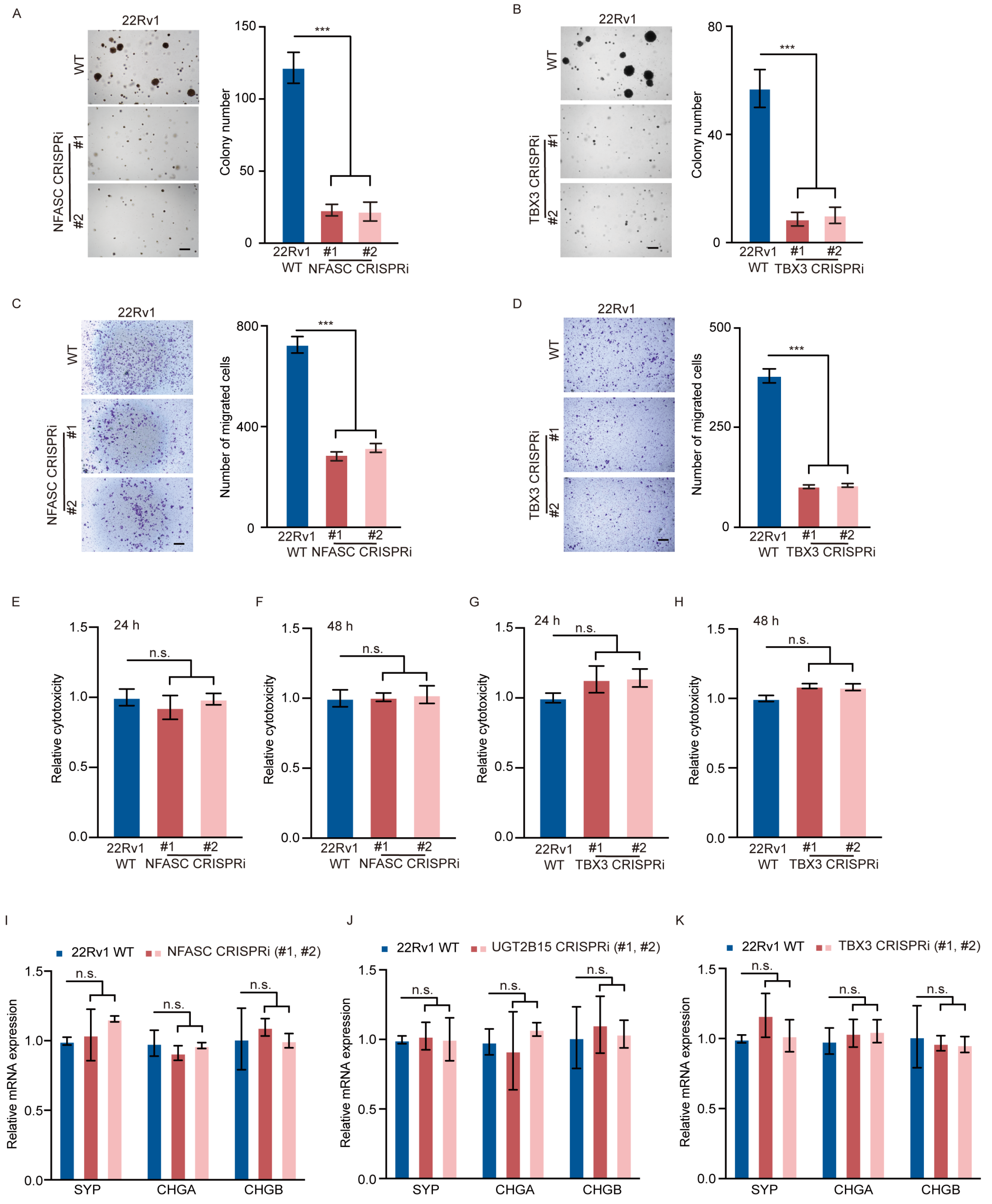


Figure S5. Knockdown of target genes partially suppresses malignant phenotypes of 22Rv1 PCa cells. (A, B) Soft agar colony formation assays in control WT cells and in cells made deficient in either *NFASC* or *TBX3* by

CRISPRi (left). Scale bar =100 μm. Quantification is at right. Data represents means ± S.E.M. of three independent experiments. ***P < 0.001. (C, D) Transwell assays in cells described above (left). Scale bar =100 μm. Cells that had migrated to lower chambers were stained with 0.1% crystal violet. Quantification is at right. Data represents means ± S.E.M. of three independent experiments. ***P < 0.001. (E-H) LDH assays in cells described above. After enzalutamide (100 μM) treatment, LDH release, was assayed as an indicator of cytotoxicity at 24 (E, G) and 48 (F, H) h. Data represent means ± S.E.M. of three independent experiments. n.s., not significant. (I-K) Real-time qPCR validation of transcript levels of NE-related genes (*SYP*, *CHGA* and *CHGB*) in WT control 22Rv1 cells and in cells made deficient in *NFASC*, *UGT2B15* and *TBX3* using CRISPRi. Data represent means ± SEM of three independent experiments. n.s., not significant.

# Supplementary Figure 6

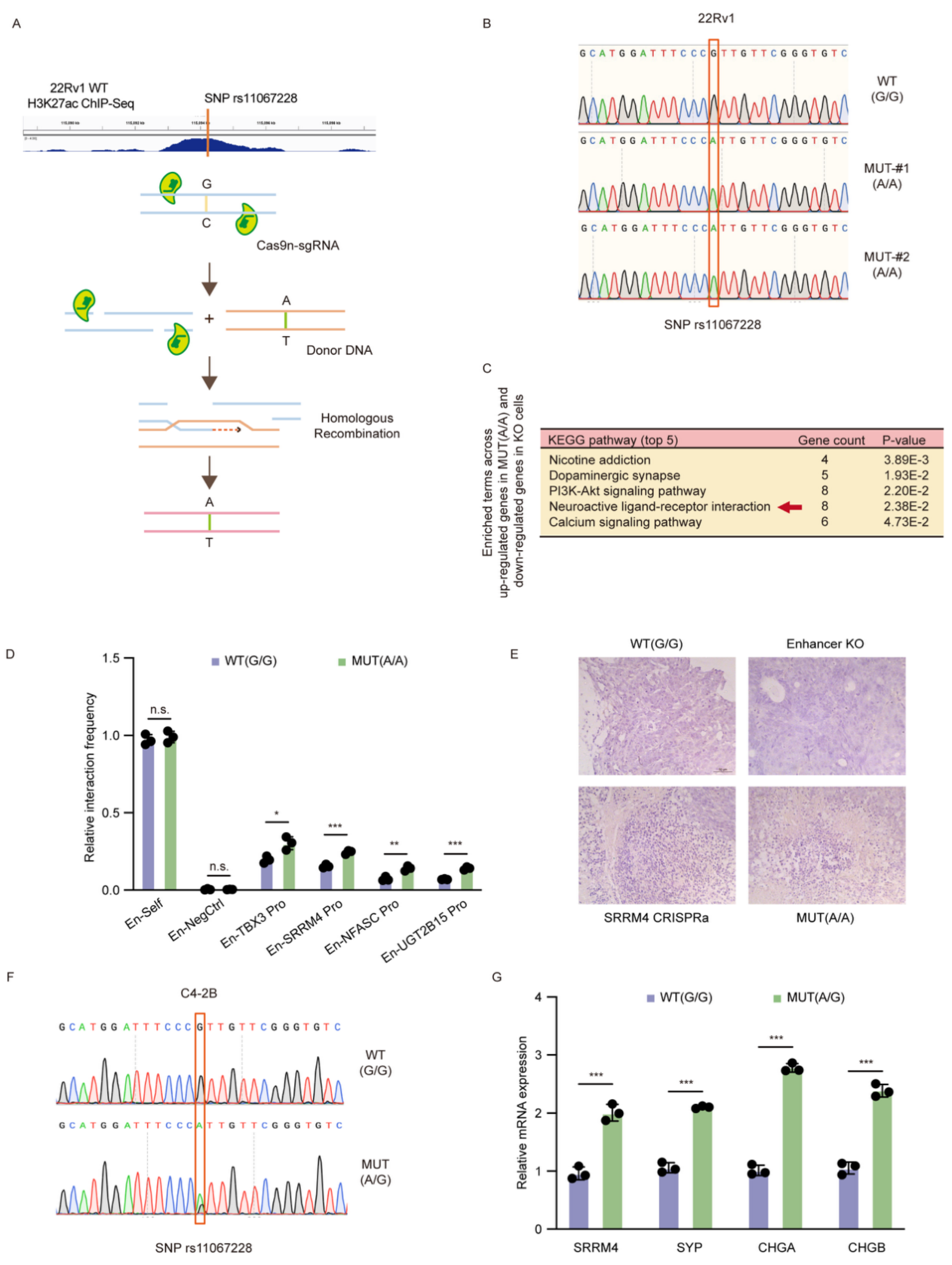


Figure S6. Functional characterization of the rs11067228 A allele across prostate cancer models. (A) Schematic showing CRISPR-Cas9-mediated mutation of SNP rs11067228 in 22Rv1 cells. (B) Sequencing analysis of

resultant SNP rs11067228 non-risk (G) and risk (A) alleles in 22Rv1 cells. (C) KEGG pathway analysis showing biological processes associated with genes overlapped between "KO vs WT" and "A vs G" in Figure 6I. (D) Quantitative 3C-qPCR analysis of enhancer-promoter interactions in WT (G/G) and MUT (A/A) cells. Data represent efficiency-corrected interaction frequencies normalized to anchor self-ligation. Data represent means ± S.E.M. of three independent experiments. ***P < 0.001, **P < 0.01, *P<0.05, n.s., not significant. (E) Representative H&E staining of indicated 22Rv1 xenografts in Figure 6M. Scale bar =50 μm. (F) Sequencing analysis of resultant SNP rs11067228 non-risk (G) and risk (A) alleles in C4-2B cells. (G) Real-time qPCR validation of transcript levels of *SRRM4* and NE-related genes (*SYP*, *CHGA* and *CHGB*) in WT (G/G) and MUT (A/G) C4-2B cells. Data represent means ± S.E.M. of three independent experiments. ***P < 0.001.

# Supplementary Figure 7

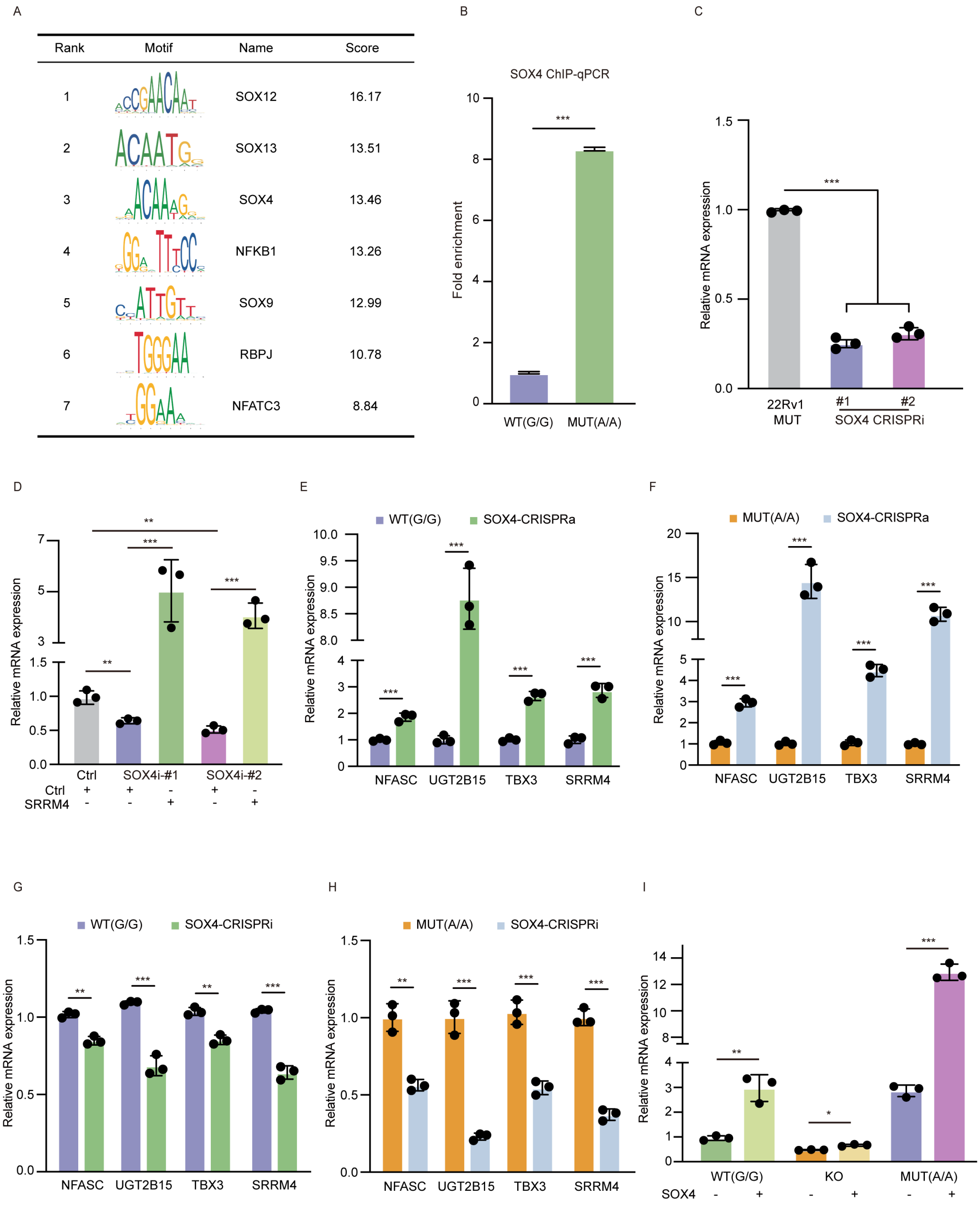


Figure S7. Identification and functional validation of SOX4 as the key transcription factor mediating rs11067228 enhancer activity. (A) Proteins binding to rs11067228 in 22Rv1 cells, based on JASPAR (an open-access

database of TF binding profiles (9th release)). (B) Results of SOX4 ChIP-qPCR performed in 22Rv1 cells homozygous for either SNP rs11067228 non-risk (G/G) or risk (A/A) alleles. Data represents means ± S.E.M. of three independent experiments. ***P < 0.001. (C) Validation of *SOX4* knockdown in 22Rv1 cells, as measured by real-time qPCR. sgRNAs targeting gene promoter regions were used in CRISPR-interference (CRISPRi) assays. Data represents means ± S.E.M. of three independent experiments. ****P* < 0.001. (D) RT-qPCR validation of mRNA level of *SRRM4* in MUT 22Rv1 cells, 2 *SOX4* knockdown lines and *SOX4*-KD cells subjected to CRISPRa to overexpress *SRRM4*. Data represents means ± S.E.M. of three independent experiments. ****P* < 0.001, **P < 0.01. (E) Transcript levels of indicated target genes before and after application of CRISPRa to overexpress SOX4 in WT(G/G) cells. Data represents means ± S.E.M. of three independent experiments. ***P < 0.001. (F) Transcript levels of indicated target genes before and after application of CRISPRa to overexpress SOX4 in MUT(A/A) cells. Data represents means ± S.E.M. of three independent experiments. ***P < 0.001. (G) Transcript levels of indicated target genes before and after application of CRISPRi to knockdown SOX4 in WT(G/G) cells. Data represents means ± S.E.M. of three independent experiments. ***P < 0.001, **P < 0.01. (H) Transcript levels of indicated target genes before and after application of CRISPRi to knockdown SOX4 in MUT(A/A) cells. Data represents means ± S.E.M. of three independent experiments. ***P < 0.001,

**P < 0.01. (I) Transcript levels of SRRM4 following CRISPRa-mediated SOX4 overexpression in 22Rv1 WT (G/G), enhancer KO, or MUT (A/A) cells. Data represents means ± S.E.M. of three independent experiments. ***P < 0.001, **P < 0.01, *P<0.05.

## **Supplementary Figure 8**

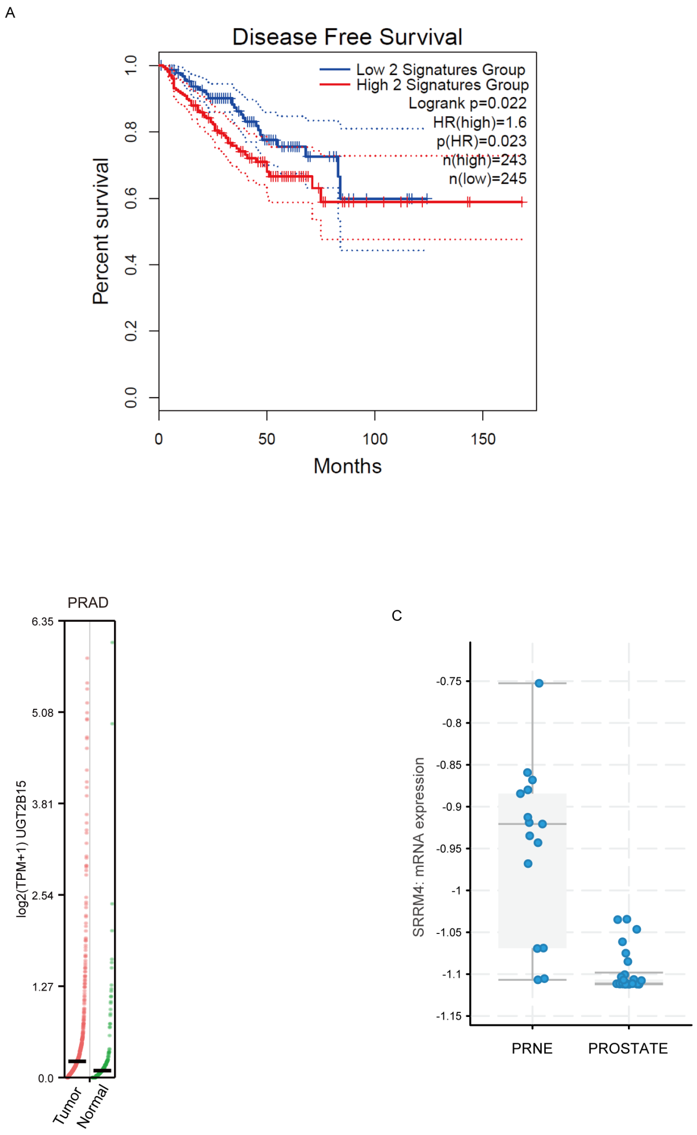


Figure S8. Combined prognostic value of *SRRM4*/*UGT2B15* and their subtype-specific expression patterns in prostate cancer. (A) Kaplan-Meier analysis of disease-free survival (DFS) in the TCGA-PRAD cohort, stratified by a combined expression score of *SRRM4* and *UGT2B15*. Patients with a high combined score exhibited significantly shorter DFS (Logrank $p < 0.05$). (B) Expression of

*UGT2B15* is significantly elevated in primary prostate adenocarcinoma compared to matched normal prostate tissue in the TCGA-PRAD dataset. (C) Expression of *SRRM4* is significantly higher in neuroendocrine prostate cancer compared to conventional prostate adenocarcinoma in a combined cohort.

Table S1. Guide RNAs and primers used to assess CRISPR/Cas9-mediated deletions.

| gRNA | Sequence |
|---|---|
| gRNA-1 | GAAAGCAGTTTCAAGACACA |
| gRNA-2 | CTCTCTAAGACCCTGCCCTG |
| Test primer-in forward | TTGGAGCTCCTGGTTCTATTTGA |
| Test primer-in reverse | GAGTCAGATATGTCTGTCCCCCT |
| Test primer-out forward | TACCTGTGCATTGGGTAGGAAG |
| Test primer-out reverse | GAAAAATCTTGCCGACTCCTGG |

Table S2. Primers used in the qRT-PCR experiment.

| Gene name | Primer | Sequence |
|---|---|---|
| TBX3 | Forward | GGACACTGGAAATGGCCGAA |
|  | Reverse | TCACCCTCGCTGGGACATAA |
| NFASC | Forward | GCACACGATCTCGGTGAGAG |
|  | Reverse | TCACGGTTTGGGTTAGGTGG |
| HSPA1B | Forward | CTTCAACATGAAGAGCGCCG |
|  | Reverse | GAGTCCCAACAGTCCACCTC |
| HSPA1A | Forward | AGCTGGAGCAGGTGTGTAAC |
|  | Reverse | CAGCAATCTTGGAAAGGCCC |

| | | |
|---|---|---|
| SRRM4 | Forward | GTTCTGGCGAGGAACCTTCA |
| | Reverse | TGCGTCGCTTGTGTTTCTTG |
| UGT2B15 | Forward | TGAGCTACTGGCTGAACTATTTAAC |
| | Reverse | TGTAGTGGGTCTTCCTAGAACT |
| MPHV2 | Forward | GTGACAGTGGCTCCTTCTAAT |
| | Reverse | GGATAGTGAGCTCCATGTTCAG |
| HPRT1 | Forward | CCTGGCGTCGTGATTAGTGAT |
| | Reverse | AGACGTTCAGTCCTGTCCATAA |
| SOX4 | Forward | CAGCAAACCAACAATGCCGA |
| | Reverse | GATCTGCGACCACACCATGA |
| SYP | Forward | ACTATGGGCAGCAAGGCTAC |
| | Reverse | AGACTGGGCACCTAGTGGAT |
| CHGB | Forward | CACGCCATTCTGAGAAGAGC |
| | Reverse | TCTCCTGGCTCTTCAAGGTG |
| CHGA | Forward | TCCAAGGCGCCAAGGA |
| | Reverse | CATCTTCAAAACCGCTGTGTTTC |

Table S3. Primers used for the 3C-PCR assay.

| Primer | Sequence |
|---|---|
| Enhancer-3C-Forward | ATGTACTTTGGAGCTCCTG |
| UGT2B15-3C-Reverse | AGGAAAAATTTCAAGTGGCAGTGT |
| NFASC-3C-Reverse | TCTGTCTGCGCAGCATTAGT |

| | |
|---|---|
| TBX3-3C-Reverse | CACAGAGGCCTTTCTCCCAG |
| SRRM4-3C-Reverse | ACATCCGTATAGCTGAGCCC |

Table S4. Guide RNAs and primers used to assess CRISPR/Cas9-mediated SNP editing.

| gRNA | Sequence |
|---|---|
| gRNA-1 Forward | CACCGTGGACACGCAAGGATTGGTG |
| gRNA-1 Reverse | AAACCACCAATCCTTGCGTGTCCAC |
| gRNA-2 Forward | CACCGAAACTTACTGTCGGAGTCCC |
| gRNA-2 Reverse | AAACGGGACTCCGACAGTAAGTTTC |
| SNP-Forward | AGGAGAGGGGCATAGTTATCCA |
| SNP-Reverse | ACACTCGACGCCCTAGCAAT |

Table S5. Primers used to construct the luciferase reporter plasmid.

| Primer | Sequence |
|---|---|
| Luc-enhancer-F | ATGGAGTGCAGCGAGGTTTAT |
| Luc-enhancer-R | GGTTGCAGGGTGTCTCATCTT |

Table S6. Primers used for 4C analysis.

| Primer | Sequence |
|---|---|
| 4C-Forward | GGGTAGTTACCTTGTTTCTGGATA |
| 4C-Reverse | CTGGTGTCCAATGACATCAAATAG |

Table S7. sgRNA sequences used in CRISPRa and CRISPRi assays.

| Primer | Sequence |
|---|---|
| sgLacZ Forward | CACCGCCCGAATCTCTATCGTGCGG |
| sgLacZ Reverse | AAACCCGCACGATAGAGATTCGGGC |
| sgUGT2B15-1a Forward | CACCGTGGCATGCACCTATTCAGAC |
| sgUGT2B15-1a Reverse | AAACGTCTGAATAGGTGCATGCCAC |
| sgUGT2B15-2a Forward | CACCGATTCAGACTGTTAGTATTAT |
| sgUGT2B15-2a Reverse | AAACATAATACTAACAGTCTGAATC |
| sgUGT2B15-3a Forward | CACCGCAGATATAAGTATGAGAAAT |
| sgUGT2B15-3a Reverse | AAACATTTCTCATACTTATATCTGC |
| sgNFASC-1a Forward | CACCGCTGTCCTGGTCCCCGCTCAG |
| sgNFASC-1a Reverse | AAACCTGAGCGGGGACCAGGACAGC |
| sgNFASC-2a Forward | CACCGTCCGCCTCTGTCCTGGTCCC |
| sgNFASC-2a Reverse | AAACGGGACCAGGACAGAGGCGGAC |
| sgNFASC-3a Forward | CACCGGAGCATCCTTCCCTCCGCCT |
| sgNFASC-3a Reverse | AAACAGGCGGAGGGAAGGATGCTCC |
| sgTBX3-1a Forward | CACCGCGCCTATGCAGCAACACAAT |
| sgTBX3-1a Reverse | AAACATTGTGTTGCTGCATAGGCGC |
| sgTBX3-2a Forward | CACCGCCAGCACTCGACCTGTGAAA |
| sgTBX3-2a Reverse | AAACTTTCACAGGTCGAGTGCTGGC |
| sgTBX3-3a Forward | CACCGATGCAGCAACACAATTGGTC |

| | |
|---|---|
| sgTBX3-3a Reverse | AAACGACCAATTGTGTTGCTGCATC |
| sgSRRM4-1a Forward | CACCGATTGTGCGAGAGACAAAACC |
| sgSRRM4-1a Reverse | AAACGGTTTTGTCTCTCGCACAATC |
| sgSRRM4-2a Forward | CACCGGCGGGCTTTTGTTATGCAGA |
| sgSRRM4-2a Reverse | AAACTCTGCATAACAAAAGCCCGCC |
| sgSRRM4-3a Forward | CACCGCCCAAGACCTGCGGGCTTTT |
| sgSRRM4-3a Reverse | AAACAAAAGCCCGCAGGTCTTGGGC |
| sgHSPA1A-1a Forward | CACCGAGGACGGGAGGCGAAAACCC |
| sgHSPA1A-1a Reverse | AAACGGGTTTTCGCCTCCCGTCCTC |
| sgHSPA1A-2a Forward | CACCGTCTGGCCTCTGATTGGTCCA |
| sgHSPA1A-2a Reverse | AAACTGGACCAATCAGAGGCCAGAC |
| sgHSPA1A-3a Forward | CACCGCATCGAGCTCGGTGATTGGC |
| sgHSPA1A-3a Reverse | AAACGCCAATCACCGAGCTCGATGC |
| sgUGT2B15-1i Forward | CACCGAAGAAGCATTGCATAAGACC |
| sgUGT2B15-1i Reverse | AAACGGTCTTATGCAATGCTTCTTC |
| sgUGT2B15-2i Forward | CACCGGGTGACTGTGTTGACATCTT |
| sgUGT2B15-2i Reverse | AAAC AAGATGTCAACACAGTCACCC |
| sgUGT2B15-3i Forward | CACCGCTGGAAGAGCTTGTTCAGAG |
| sgUGT2B15-3i Reverse | AAACCTCTGAACAAGCTCTTCCAGC |
| sgNFASC-1i Forward | CACCGTGGTCTCTGCCCTAATGCGG |
| sgNFASC-1i Reverse | AAACCCGCATTAGGGCAGAGACCAC |

| | |
|---|---|
| sgNFASC-2i Forward | CACCGGCAGCGGACAGCTCGGACAG |
| sgNFASC-2i Reverse | AAACCTGTCCGAGCTGTCCGCTGCC |
| sgNFASC-3i Forward | CACCGTAATGCGGCGGCTGGCGGCG |
| sgNFASC-3i Reverse | AAACCGCCGCCAGCCGCCGCATTAC |
| sgTBX3-1i Forward | CACCGACCTTCTAGAGCCGCCGAGC |
| sgTBX3-1i Reverse | AAACGCTCGGCGGCTCTAGAAGGTC |
| sgTBX3-2i Forward | CACCGGAGAAGAGCCCAGCAAGATT |
| sgTBX3-2i Reverse | AAACAATCTTGCTGGGCTCTTCTCC |
| sgTBX3-3i Forward | CACCGGAAACCGAGACACCCTCCGG |
| sgTBX3-3i Reverse | AAACCCGGAGGGTGTCTCGGTTTCC |
| sgSRRM4-1i Forward | CACCGCCGCCCTGAACTCCGATCTC |
| sgSRRM4-1i Reverse | AAACGAGATCGGAGTTCAGGGCGGC |
| sgSRRM4-2i Forward | CACCGTCTCTGGGTTTCACCCGGAC |
| sgSRRM4-2i Reverse | AAACGTCCGGGTGAAACCCAGAGAC |
| sgSRRM4-3i Forward | CACCGTGAACTCCGATCTCTCCCAC |
| sgSRRM4-3i Reverse | AAACGTGGGAGAGATCGGAGTTCAC |
| sgSOX4-1a Forward | CACCGTGAAAGGATAAAGAGGCGCG |
| sgSOX4-1a Reverse | AAACCGCGCCTCTTTATCCTTTCAC |
| sgSOX4-2a Forward | CACCGGGTTTGGCATGAGGAAGCGT |
| sgSOX4-2a Reverse | AAACACGCTTCCTCATGCCAAACCC |
| sgSOX4-3a Forward | CACCGGCATCGGGTTCCAAGCCAAT |

| | |
|---|---|
| sgSOX4-3a Reverse | AAACATTGGCTTGGAACCCGATGCC |
| sgSOX4-1i Forward | CACCGCGCTCTTTAAGAGTCTGCAC |
| sgSOX4-1i Reverse | AAACGTGCAGACTCTTAAAGAGCGC |
| sgSOX4-2i Forward | CACCGGCAAGAGAAACTGTGTGTGA |
| sgSOX4-2i Reverse | AAACTCACACACAGTTTCTCTTGCC |

Table S8. Sequences of biotinylated double-strand oligonucleotides used in pull-down assays.

| Primer | Sequence |
|---|---|
| Biotin SNP-A Forward | GGATTTCCCATTGTTCGGGTG |
| SNP-A Reverse | CACCCGAACAATGGGAAATCC |
| Biotin SNP-G Forward | GATTTCCCGTTGTTCGGGTGT |
| SNP-G Reverse | ACACCCGAACAACGGGAAATC |

Table S9. 3C-qPCR primer sequences and amplification efficiencies

| Primer Pair | Sequence (5'-3') | Efficiency (%) | Slope |
|---|---|---|---|
| Enhancer-F/ TBX3-Pro-R | TCCCATTGTTCGGGTGTCTG/ GAGGCTCCAACTGCTGACTT | 98.3 | -3.36 |
| Enhancer-F/ SRRM4-Pro-R | GGGAGGTCTGGGGGTAGTTA/ ACAACTCTGTTGGGGGTCTC | 96 | -3.42 |

| | | | |
|---|---|---|---|
| Enhancer-F/ NFASC-Pro-R | TTGGAGCTCCTGGTTCTATTTGA/ GCAGGGGACTCACCAAATGTT | 102.6 | -3.26 |
| Enhancer-F/ UGT2B15-Pro-R | TGGGCTGCCGCAGTCTATAA/ ACCAAGGATCAAGGGACTAGC | 106.4 | -3.18 |
| Enhancer-F/ TBX3-Up-R | TGTGCGGGATCTGCCTTATC/ GTGATCCCACCACGGATTCA | 108.7 | -3.13 |
| Enhancer-F/ TBX3-Dn-R | GCTTAAGCTTTGGGACCCTG/ AGTGCCCTGGTCCAAACAAA | 95.5 | -3.44 |
| Enhancer-F/ SRRM4-Up-R | TGTGCGGGATCTGCCTTATC/ TACCAAGCTGGTCGTATGCC | 106.7 | -3.17 |
| Enhancer-F/ SRRM4-Dn-R | GCGGGATCTGCCTTATCTCC/ AACATCTGGCTGCTCCTTCC | 100.5 | -3.31 |
| Enhancer-F/ NFASC-Up-R | CATGGATTTCCCGTTGTTCG/ AGACTGGAGTGATTTGAGCCC | 92 | -3.53 |
| Enhancer-F/ NFASC-Dn-R | CTGGGCTGCCGCAGTCTATAA/ AGGAGGGTGTCGAGTCTGG | 109.3 | -3.12 |
| Enhancer-F/ UGT2B15-Up-R | CCGTTGTTCGGGTGTCTGTG/ AAGGAGGGTGTCGAGTCTGG | 104.1 | -3.23 |
| Enhancer-F/ UGT2B15-Dn-R | ATTTCCCGTTGTTCGGGTGT/ GGAGATAAGGCAGATCCCGC | 100.4 | -3.31 |

| | | | |
|---|---|---|---|
| Enhancer-Up-F/ TBX3-R | GGGGATAGGAAGAAAGCTTATGTA/ CAGACACCCGAACAATGGGA | 98 | -3.37 |
| Enhancer-Dn-F/ TBX3-R | GGTGATTCTCAGGCCCCAAA/ TAGTAGGTGCCTCCCACCTC | 105.5 | -3.2 |
| Enhancer-Up-F/ SRRM4-R | GGGGATAGGAAGAAAGCTTGGATT/ TGCTGTAGCGTGGGCTTTAG | 91.1 | -3.56 |
| Enhancer-Dn-F/ SRRM4-R | GGACTCCCTTGTAGCTCGTT/ AGGAATCCAAGCTTCCTACAAT | 100.5 | -3.31 |
| Enhancer-Up-F/ NFASC-R | GAGCTACTGTGAGGCTGATTTC/ TGGTGGCCCAGTTACCCATT | 110.6 | -3.09 |
| Enhancer-Dn-F/ NFASC-R | AAGGGGACTCCCTTGTAGCTC/ CAAGTCTGAGATGGCTGGATGC | 102.8 | -3.26 |
| Enhancer-Up-F/ UGT2B15-R | GTTAGCCAACAGGTAGGAGCTA /GGCGGGATATGAAATTCTGG | 98.2 | -3.37 |
| Enhancer-Dn-F/ UGT2B15-R | GGTGATTCTCAGGCCCCAAA/ TAGTAGGTGCCTCCCACCTC | 92.7 | -3.51 |
| Enhancer-F/ Self-R | ATGGACACGCAAGGATTGGT/ CAGACACCCGAACAATGGGA | 97.6 | -3.38 |
| Enhancer-F/ NegCtrl-R | TGTGCCGACCAGGAAAGAAG/ TTTGTGCGCCACAACCAAGTCC | 100.8 | -3.3s |

Table S10. Key resources table

| REAGENT or RESOURCE | SOURCE | IDENTIFIER |
|---|---|---|
| Antibodies | | |
| Rabbit anti-H3K27ac | Abcam | Cat# ab4729 RRID: AB_2118291 |
| Rabbit anti-UGT2B15 | Abcam | Cat# ab154864 RRID: AB_2722651 |
| Rabbit anti-Neurofascin | Abcam | Cat# ab31457 RRID: AB_881185 |
| Rabbit anti-TBX3 | Abcam | Cat# ab99302 RRID: AB_10861059 |
| Rabbit anti-SRRM4 | ThermoFisher | Cat# PA5-112589 RRID: AB_2867324 |
| Rabbit anti-HSPA1A | ProteinTech | Cat# 10995-1-AP RRID: AB_2264230 |
| Rabbit anti-Synaptophysin | Abcam | Cat# ab32127 RRID: AB_2286949 |
| Rabbit anti-Chromogranin A | Abcam | Cat# ab283265 |
| Rabbit anti-Chromogranin B | Abcam | Cat# ab12242 RRID: AB_298965 |

| | | |
|---|---|---|
| Rabbit anti-beta Tubulin | Biyotime | Cat# AF1216 RRID: AB_2924787 |
| Goat anti-rabbit IgG H&L | Abcam | Cat# ab6702 RRID: AB_956012 |
| Rabbit anti-SOX4 | Abcam | Cat# ab86809 RRID: AB_10714562 |
| Chemicals, Peptides, and Recombinant Proteins | | |
| Fetal Bovine Serum (FBS) | VivaCell | Cat# C04001-500 |
| Penicillin and Streptomycin | Gibco | Cat# 15140-122 |
| Triton X-100 | Sigma-Aldrich | Cat# T9284 |
| HEPES | Solarbio | Cat# H1095 |
| EDTA, FREE ACID | Solarbio | Cat# E8040 |
| EDTA $Na_2$ | Solarbio | Cat# E8030 |
| EGTA | Solarbio | Cat# E8050 |
| Deoxycholic acidsodium salt | Solarbio | Cat# D8330 |
| Nonidet P-40 | Solarbio | Cat# N8030 |
| Tween-20 | Solarbio | Cat# T8220 |
| SDS | Solarbio | Cat# S8010 |

| | | |
|---|---|---|
| Protein A Magnetic Beads | ThermoFisher | Cat# 10002D |
| Protein G Magnetic Beads | ThermoFisher | Cat# 10004D |
| Puromycin | MedChemExpress | Cat# HY-B1743A |
| Blasticidin | Solarbio | Cat# B9300 |
| Hygromycin | Solarbio | Cat# H8080 |
| DpnII | New England Biolabs | Cat# R0543L |
| CviqI | New England Biolabs | Cat# R0639L |
| HindIII-HF | New England Biolabs | Cat# R3014L |
| BsmbI-v2 | New England Biolabs | Cat# R0739L |
| Ampicillin | Solarbio | Cat# A8180 |
| Formaldehyde solution | Sigma-Aldrich | Cat# F8775 |
| Glycine | Solarbio | Cat# G8200 |
| Streptavidin magnetic C1 beads | ThermoFisher | Cat# 65002 |

| | | |
|---|---|---|
| Crystal violet | Solarbio | Cat# G1063 |
| RNase A | Solarbio | Cat# R1030 |
| Matrigel | Corning | Cat# 356234 |
| SeaPlaque$^{TM}$Agarose | Lonza | Cat# 50101 |
| TRIzol | ThermoFisher | Cat# 15596018 |
| PMSF | Sigma-Aldrich | Cat# P7626-25G |
| Complete EDTA-free Protease Inhibitor Cocktail | Roche | Cat# 11836170001 |
| T4 DNA Ligase | Takara | Cat# 2011A |
| Proteinase K | CMBio | Cat# CW2298M |
| Dual-Luciferase Reporter Assay System kit | Promega | Cat# E1910 |
| CytoTox 96® Non-Radioactive Cytotoxicity Assay | Promega | Cat# G1780 |
| PrimeScript RT reagent with a gDNA eraser Kit | Takara | Cat# RR047B |
| qPCR SYBR Green Master Mix | YEASEN | Cat# 11201ES03 |

| | | |
|---|---|---|
| Premix Ex-Taq HS | Takara | Cat# RR030 |
| Lipofectamine 3000 | ThermoFisher | Cat# L3000-015 |
| Falcon Cell Culture Inserts | Corning | Cat# 353097 |
| Deposited Data | | |
| H3K27ac ChIP-seq | This paper | GSE274154 |
| 4C-seq | This paper | GSE274153 |
| RNA-seq | This paper | GSE274155 |
| Mass spectrometry proteomics | This paper | PXD055628 |
| Experimental Models: Cell Lines | | |
| 22Rv1 | ATCC | Cat# CRL-2505 |
| LNCaP | ATCC | Cat# CRL-1740 |
| C4-2B | ATCC | Cat# CRL-3315 |
| HEK-293T | ATCC | Cat# CRL-3216 |
| Recombinant DNA | | |
| pGL3 Promoter vector | Promega | Cat# E1761 |
| pGL3 Basic vector | Promega | Cat# E1751 |
| pRL-TK Renilla luciferase vector | Promega | Cat# E2241 |

| | | |
|---|---|---|
| pSpcas9n(sgRNAs) | Addgene | Cat# 62987 |
| pMD2.G | Addgene | Cat# 12259 |
| psPAX2 | Addgene | Cat# 12260 |
| lenti-dCas9-KRAB-blast | Addgene | Cat# 89567 |
| lentiGuide-Puro | Addgene | Cat# 52963 |
| lentiMPHv2 | Addgene | Cat# 89308 |
| lentiSAMv2 | Addgene | Cat# 75112 |
| lentiCRISPR v2 | Addgene | Cat# 52961 |
| Software and Algorithms | | |
| GraphPad Prism9 | GraphPad software | https://www.graphpad.com |
| ImageJ | NIH | https://imagej.nih.gov/ij |
| BWA | Li and Durbin, 2009 (58) | https://github.com/lh3/bwa |
| HTseq (v0.11.3) | Anders et al., 2015 (61) | https://github.com/simon-anders/htseq |
| DEseq2 (v1.38.3) | Love et al., 2014 (62) | https://bioconductor.org/packages/release/bioc/html/DESeq2.html |
| DAVID | Huang da et al., 2009 (63) | https://david-d.ncifcrf.gov/ |

| | | |
|---|---|---|
| HISAT2 | Kim et al., 2019 (55) | https://github.com/DaehwanKimLab/hisat2 |
| R (version 4.0.3) | R Core Team, 2017 | https://www.R-project.org/ |
| RStudio (version 2021.09.1) | RStudio Team, 2016 | https://www.rstudio.com/ |
| GPP Web Portal – sgRNA design | Broad institute | https://portals.broadinstitute.org/gpp/ public/analysis-tools/sgrna-design |
